# Supplementary material for: Upregulation of angiotensin-(1–7) formation in human podocytes – enzyme activity assay upon fluid flow shear stress
Source: PLoS One. 2026 Jan 9;21(1):e0339874. doi: 10.1371/journal.pone.0339874 (PMC12788633; doi:10.1371/journal.pone.0339874)
Supplement: S1 Table — (PDF) [file pone.0339874.s001.pdf]

Supplement Table 1: Primer sequences for genes of interest.

Gene	Ensembl no.	Sense Primer (5'-3')	Antisense Primer (5'-3')	Amplicon
ACE	ENSG00000159640	CCGGCAACTTTTCTGCTGAC	TTAGCAGGGCGTTGTACTG	327
ACE2	ENSG00000130234	CAATGGACGACTTCCTGAC	CCCACCACTTTTTCATCCAC	342
AGT	ENSG00000135744	TTCTCGGTGACTCAAGTGC	GTTGGGTAGACTCTGTGGG	349
AGTR1	ENSG00000144891	AGCACTGGCTGACTTATGCT	CTGCCAGCAGCCAAATGAT	260
AGTR2	ENSG00000180772	TACCAGCGGTCTTCACTTCG	CACAGGTCCAAAGAGCCAG	304
GUSB	ENSG00000169919	TACGAACGGGAGGTGATCC	TGGCGATAGTGATTCGGAG	214
PRCP	ENSG00000137509	GAAAATCAACCTGTCATTGC	CACCATTGCCAGATTCACC	364
PREP	ENSG00000085377	ACATATCCATCACACCCAAC	TCCGAGCACCCATAATCAG	380
REN	ENSG00000143839	CAAGAGAATGCCCTCAATC	CCCTTGGGAGATGATGTTG	544

ACE, angiotensin I converting enzyme; *ACE2*, angiotensin converting enzyme 2; *AGT*, angiotensinogen; *AGTR1*, angiotensin II receptor type 1; *AGTR2*, angiotensin II receptor type 2; *GUSB*, glucuronidase beta; *PRCP*, prolylcarboxypeptidase; *PREP*, prolyl endopeptidase; *REN*, renin; bp, base pairs. Primers were designed using Primer-BLAST [1].

References

1. Ye J, Coulouris G, Zaretskaya I, Cutcutache I, Rozen S, Madden TL. Primer-BLAST: A tool to design target-specific primers for polymerase chain reaction. *BMC Bioinformatics.* 2012;**13**(1).
