# Supplementary material for: Upregulation of angiotensin-(1–7) formation in human podocytes – enzyme activity assay upon fluid flow shear stress
Source: PLoS One. 2026 Jan 9;21(1):e0339874. doi: 10.1371/journal.pone.0339874 (PMC12788633; doi:10.1371/journal.pone.0339874)
Supplement: S1 File — (PDF) [file pone.0339874.s002.pdf]

Supplement Methods

PCR

Components of the angiotensin system were determined on RNA level in differentiated hPC by polymerase chain reaction (PCR). We analyzed gene expression of angiotensin I converting enzyme (*ACE*), angiotensin converting enzyme 2 (*ACE2*), angiotensinogen (*AGT*), angiotensin II receptor type 1 (*AGTR1*), angiotensin II receptor type 2 (*AGTR2*), prolylcarboxypeptidase (*PRCP*), prolyl endopeptidase (*PREP*), and renin (*REN*). Primer sequences are listed in Supplement Table 1. cDNA of hPCs were amplified using the C1000 Touch™ Thermal Cycler. cDNA from different human organs served as positive controls as indicated. Samples and 100bp DNA ladder (cat. no. N3231L, New England BioLabs, Ipswich, MA, US) were applied on 2% agarose gels and amplicons detected using RedSafe™ (cat. no. 21141, iNtRON Biotechnology, South Korea) with the FastGene® FAS-V Imaging System (cat. no. GP-FAS-V, NIPPON Genetics EUROPE, Düren, Germany). Gel images are shown in Supplement Figure 1.

Western Blot

ACE2 and PRCP were additionally detected on protein level by western blot. 20µg of protein was used for hPC samples and 10µg protein for human kidney samples. 0,001 µg recombinant ACE2 (cat. no. TP720353, OriGene, Rockville, Maryland, US) was applied and 0,02 µg recombinant PRCP (cat. no. 7164-SE, R&D Systems, Minneapolis, MN, US). Protein samples were normalized to 30µl using RIPA-buffer with protease and phosphatase inhibitors and were mixed with 8µl loading buffer, denatured at 95 °C for 5 min, and stored on ice. Samples were applied on a Novex™

WedgeWell™ 4 bis 20 %, Tris-Glycin, 1,0 mm, Mini-Protein-Gel (cat. no. XP04202BOX, Thermo Fisher Scientific, Waltham, MA, US) and gel electrophoresis was conducted with a XCell SureLock Mini-Cell (cat. no. EI0001, Thermo Fisher Scientific) for 80 min at 150V. Proteins were blotted on a nitrocellulose membrane (cat. no. LC2001, Thermo Fisher Scientific). ACE2 was detected using rabbit anti-ACE2 antibody (anti-ACE2-1, cat. no. sc-20998, Santa Cruz Biotechnology, Figure S4A) diluted 1:200 and additionally with rabbit anti-ACE2 antibody (anti-ACE2-2, cat. no. PA5-85139, Thermo Fisher Scientific, Figure S4B) diluted 1:300. PRCP was detected using rabbit anti-PRCP (cat. no. ATA-HPA017065, biozol, Hamburg, Germany, Figure S4C) diluted 1:1000. Primary antibodies were incubated over night at 4°C. Following washing, blots were then incubated for 1h at room temperature with horseradish peroxidase conjugated goat anti-rabbit (cat. no. sc-2004, Santa Cruz Biotechnology) diluted 1:5000 as the second antibody. GAPDH was detected using primary antibody rabbit-anti GAPDH (TA346868, OriGene) diluted 1:10000 and secondary antibody horseradish peroxidase conjugated goat anti-rabbit (cat. no. sc-2004, Santa Cruz Biotechnology) diluted 1:5000. Staining were detected using SuperSignal™ West Dura Extended Duration Substrate (cat no. 3707, Thermo Fisher Scientific) with the Fusion Fx7 (peqlab Biotechnologie GmbH, Erlangen, Germany).
