# Supplementary material for: Upregulation of angiotensin-(1–7) formation in human podocytes – enzyme activity assay upon fluid flow shear stress
Source: PLoS One. 2026 Jan 9;21(1):e0339874. doi: 10.1371/journal.pone.0339874 (PMC12788633; doi:10.1371/journal.pone.0339874)
Supplement: S1 Fig — (PDF) [file pone.0339874.s003.pdf]

S1 Fig

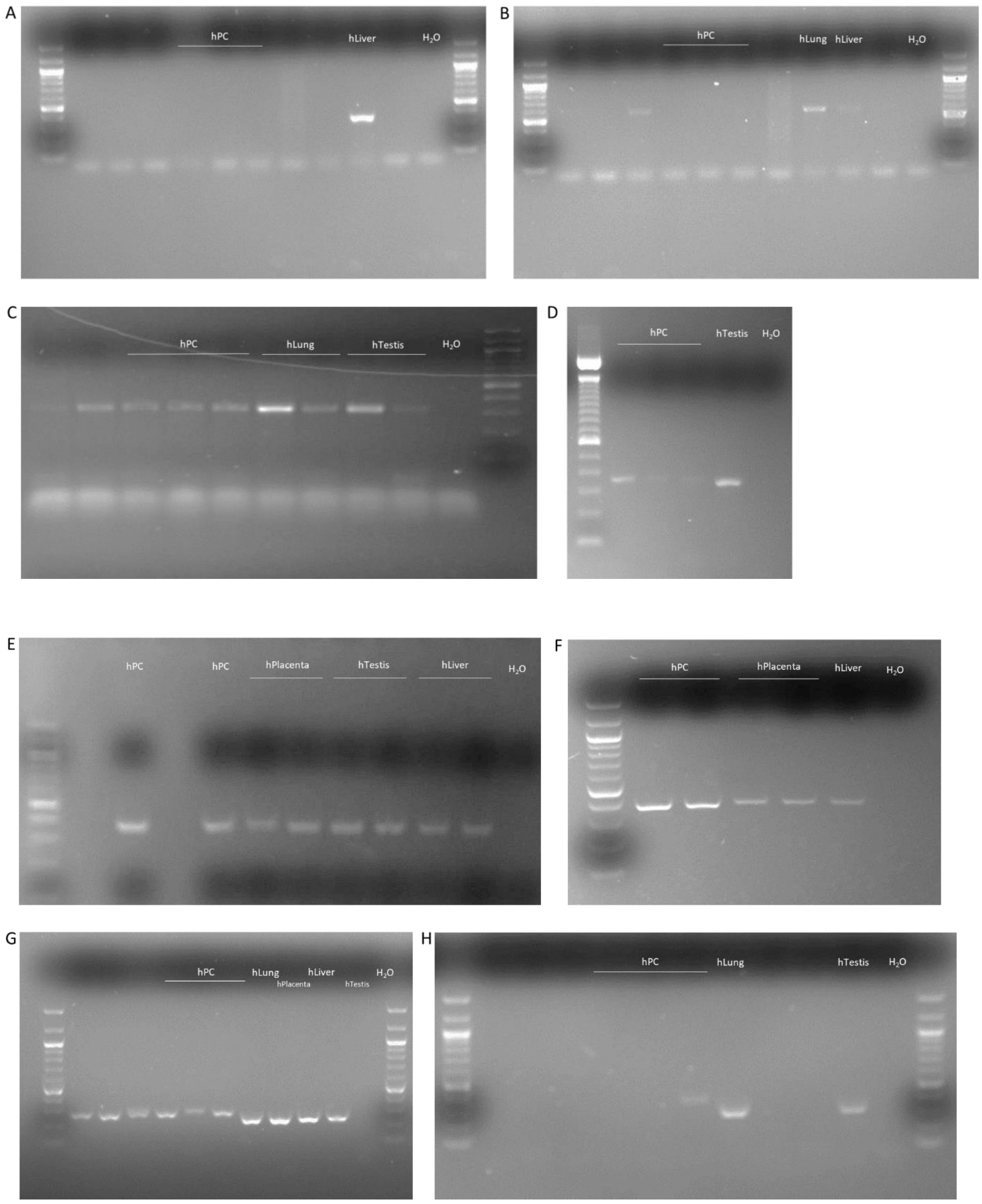

S1 Fig. Characterization of angiotensin system components on RNA level in differentiated hPC. (A) No *AGT* and **(B)** no renin (*REN*) gene expression was detected in

hPC. **(C)** *ACE*, **(D)** *ACE2*, **(E)** *PRCP*, **(F)** *PREP*, and **(G)** *AGTR1* gene expression were seen in hPC. **(H)** *AGTR2* was only weakly expressed in one of three differentiated hPC samples.
