# Supplementary material for: Upregulation of angiotensin-(1–7) formation in human podocytes – enzyme activity assay upon fluid flow shear stress
Source: PLoS One. 2026 Jan 9;21(1):e0339874. doi: 10.1371/journal.pone.0339874 (PMC12788633; doi:10.1371/journal.pone.0339874)
Supplement: S2 Fig — (PDF) [file pone.0339874.s004.pdf]

S2 Fig

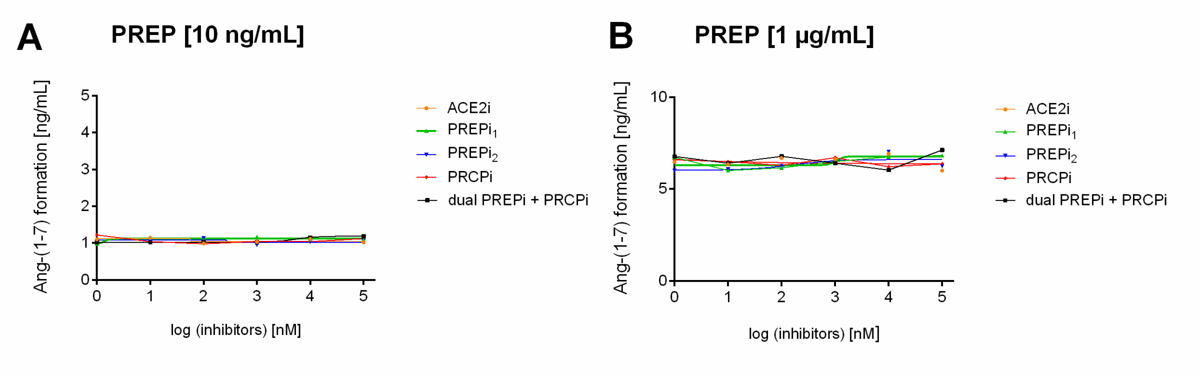

S2 Fig. Inhibitor effects on PREP-mediated Ang II to Ang-(1-7) conversion. Ang II to Ang-(1-7) conversion by PREP 10 ng/mL (A) and 1µg/mL (B) was not detected. Thus, none of the five tested inhibitors showed an effect.
