# Supplementary material for: Upregulation of angiotensin-(1–7) formation in human podocytes – enzyme activity assay upon fluid flow shear stress
Source: PLoS One. 2026 Jan 9;21(1):e0339874. doi: 10.1371/journal.pone.0339874 (PMC12788633; doi:10.1371/journal.pone.0339874)
Supplement: S3 Fig — (PDF) [file pone.0339874.s005.pdf]

S3 Fig

A **ACE2 [10 ng/mL]**

Inhibitor	log (IC_{50}) [nM]	IC_{50} [nM]	R^2
ACE2i	2.810	646.0	0.9997
$PREPi_1$	n.a.	n.a.	n.a.
$PREPi_2$	~ 3.102	1266	0.1892
PRCPi	~ 4.155	~ 14275	0.4815
dual PREPi + PRPCi	~ 1.022	~ 10.51	0.5074

B **PRCP [10 ng/mL]**

Inhibitor	log (IC_{50}) [nM]	IC_{50} [nM]	R^2
ACE2i	n.a.	n.a.	n.a.
$PREPi_1$	~ 4.485	~ 30552	0.9175
$PREPi_2$	3.921	8336	0.9779
PRCPi	~ -0.6062	~ 0.2476	0.9986
dual PREPi + PRPCi	2.359	228.4	0.9994

C **PREP [10 ng/mL]**

Inhibitor	log (IC_{50}) [nM]	IC_{50} [nM]	R^2
ACE2i	~ 1.753	~ 56.60	0.4908
$PREPi_1$	~ 0.1407	~ 1.383	0.6867
$PREPi_2$	~ 2.445	~ 278.9	0.3505
PRCPi	n.a.	n.a.	n.a.
dual PREPi + PRPCi	3.725	5305	0.9974

D **PREP [1 µg/mL]**

Inhibitor	log (IC_{50}) [nM]	IC_{50} [nM]	R^2
ACE2i	n.a.	n.a.	n.a.
$PREPi_1$	~ 3.025	~ 1058	0.4931
$PREPi_2$	2.226	168.3	0.5299
PRCPi	n.a.	n.a.	n.a.
dual PREPi + PRPCi	n.a.	n.a.	n.a.

S3 Fig. Inhibition of Ang-(1-7) formation from substrate Ang II by different inhibitors. Log (IC_{50}), IC_{50} and R^2 calculated for the respective inhibitor graphs (Figure 4 A, C, E, F) are listed in the tables. For each tested enzyme, inhibition of enzyme activity was tested for the inhibitors ACE2i, PRCPi, $PREPi_1$, $PREPi_2$, and dual PREPi + PRCPi. **(A)** Inhibition of ACE2 activity: Only the specific ACE2i exhibited inhibitory effects and calculations were accurate with an R^2 = 0.9997. **(B)** Inhibition of PRCP activity: PRCP was inhibited by PRCPi > dual PREPi + PRCPi > $PREPi_2$. $PREPi_1$ exhibited only a small inhibitory effect and the R^2 of 0.9175 indicated that the calculation is less accurate. ACE2i had no inhibitory effect on PRCP. **(C)** Inhibition of PREP (10 ng/mL) and **(D)** PREP (1 µg/mL) activity: Since no conversion of Ang II to Ang-(1-7) by PREP was observed, no inhibitory effects could be determined, reflected by inaccurate R^2 of the calculations. (n.a. not applicable).
