# Supplementary material for: Upregulation of angiotensin-(1–7) formation in human podocytes – enzyme activity assay upon fluid flow shear stress
Source: PLoS One. 2026 Jan 9;21(1):e0339874. doi: 10.1371/journal.pone.0339874 (PMC12788633; doi:10.1371/journal.pone.0339874)
Supplement: S4 Fig — (PDF) [file pone.0339874.s006.pdf]

S4 Fig

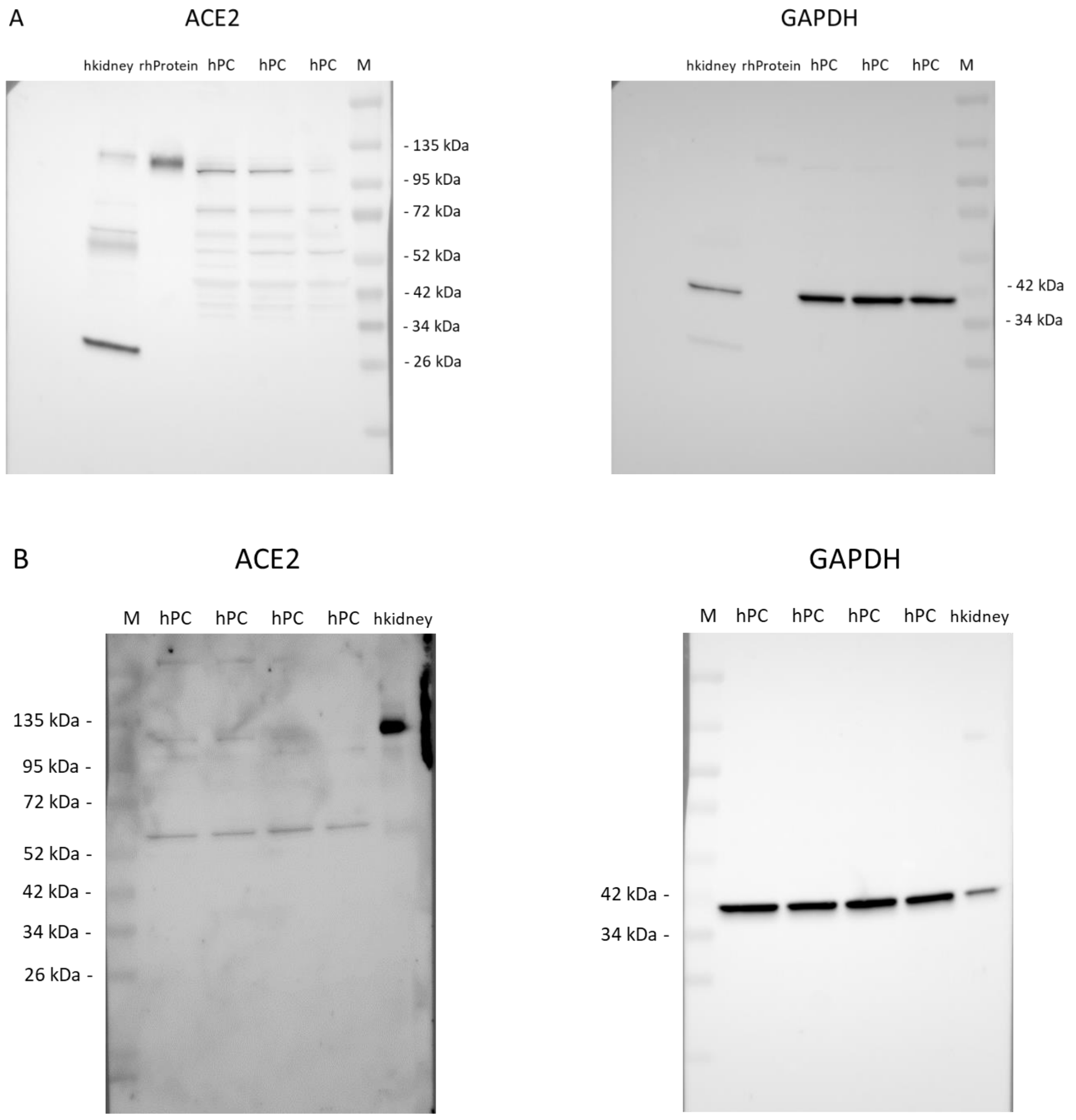
A
ACE2
hkidney rhProtein hPC hPC hPC M
- 135 kDa
- 95 kDa
- 72 kDa
- 52 kDa
- 42 kDa
- 34 kDa
- 26 kDa
GAPDH
hkidney rhProtein hPC hPC hPC M
- 42 kDa
- 34 kDa
B
ACE2
M hPC hPC hPC hPC hkidney
135 kDa -
95 kDa -
72 kDa -
52 kDa -
42 kDa -
34 kDa -
26 kDa -
GAPDH
M hPC hPC hPC hPC hkidney
42 kDa -
34 kDa -

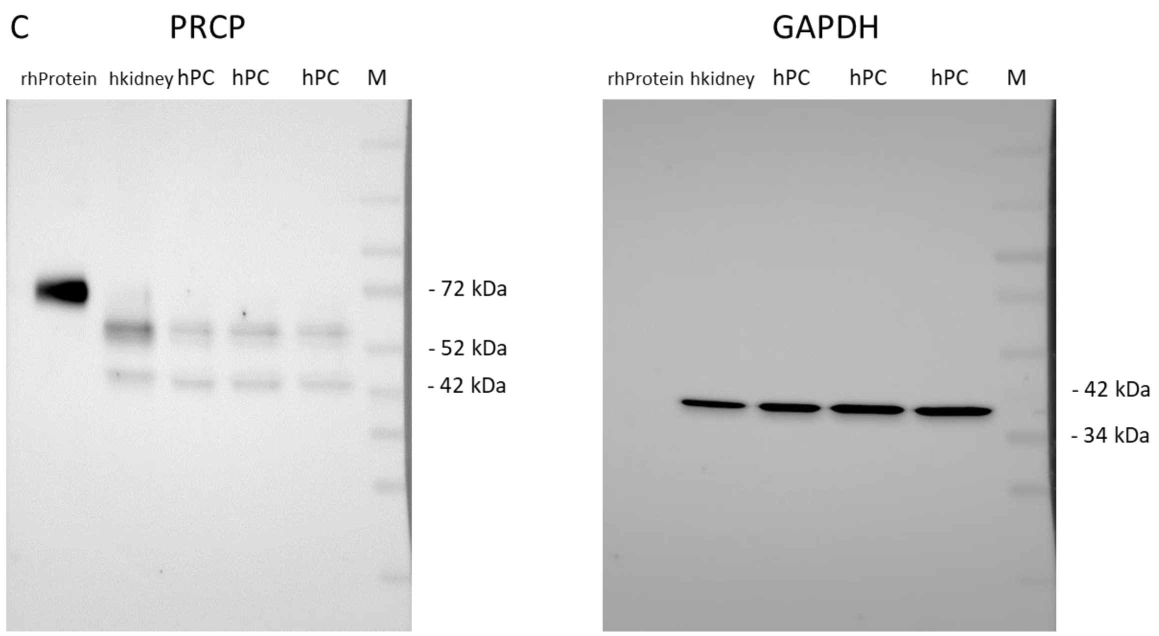

S4 Fig: Western blot analyses of (A, B) ACE2 and (C) PRCP. (A) Using anti-ACE2-1, glycosylated full-length ACE2 (120 kDa) and a short ACE2 isoform (52 kDa) was detected in human kidney (10µg, positive control) and confirmed in hPC samples (20 µg) as well. Recombinant human ACE2 was detected at 120 kDa. (B) Using anti-ACE2-2, glycosylated full-length ACE2 (120 kDa) was clearly detected in human kidney and weak signals were detected in hPC samples. Short-ACE2 isoform (52 kDa) was detected in hPC samples. In contrast, human kidney only showed a faint band. (C) Human kidney as well as hPC samples revealed the same PRCP pattern (55kDa and 45 kDA). Recombinant human PRCP was detected at 70 kDa, as described by the manufacturer. (A-C) GAPDH (37kDa) detection served as loading control for human kidney and hPC samples.
