# Supplementary material for: Upregulation of angiotensin-(1–7) formation in human podocytes – enzyme activity assay upon fluid flow shear stress
Source: PLoS One. 2026 Jan 9;21(1):e0339874. doi: 10.1371/journal.pone.0339874 (PMC12788633; doi:10.1371/journal.pone.0339874)
Supplement: S5 Fig — (PDF) [file pone.0339874.s007.pdf]

S5 Fig

		Ang 1-7 [pg/ml]	
	**solvent**	**ZPP**	**ZPP/LBQ**
**NEP_QC (100ng/ml)_1-1**	36257	35206	823
**NEP_QC (100ng/ml)_1-2**	34030	38142	621
**PREP_QC (100ng/ml)_1-1**	7445	314	324
**PREP_QC (100ng/ml)_1-2**	7479	237	308

S5 Fig: Results NEP assay. NEP- and PREP-derived Ang-(1-7) formation from substrate Ang I was detected by LC-MS/MS, verifying activity of the recombinant PREP enzyme used for inhibitor tests (Fig S2). Dual PREP and PRCP inhibitor reduced PREP-derived Ang I to Ang-(1-7) conversion, but did not affect Ang-(1-7) formation by NEP. NEP inhibitor LBQ inhibited Ang-(1-7) formation by NEP.
