# Supplementary material for: Upregulation of angiotensin-(1–7) formation in human podocytes – enzyme activity assay upon fluid flow shear stress
Source: PLoS One. 2026 Jan 9;21(1):e0339874. doi: 10.1371/journal.pone.0339874 (PMC12788633; doi:10.1371/journal.pone.0339874)
Supplement: S6 Fig — (PDF) [file pone.0339874.s008.pdf]

S6 Fig

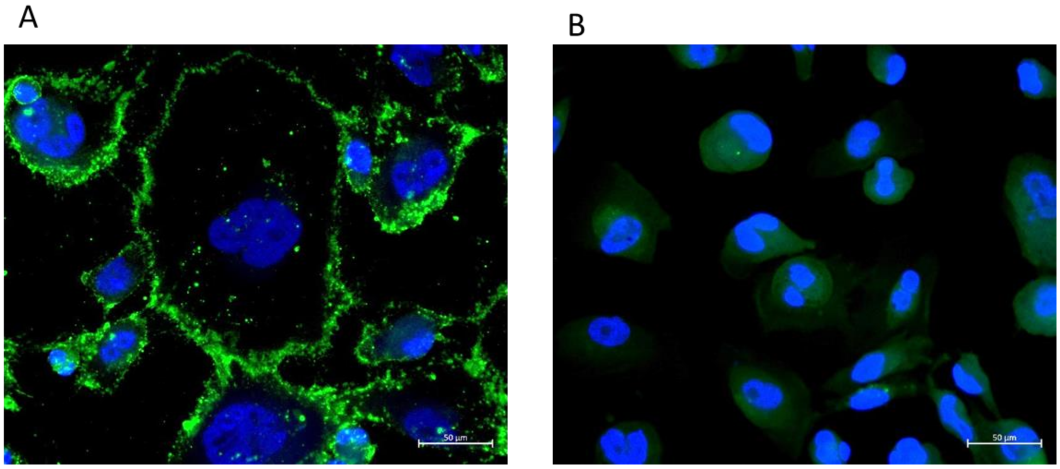

S6 Fig. Representative immunofluorescence image of MAS1. (A) MAS1 antibody (cat. no. PA5-97953, Thermo Fisher Scientific) was diluted 1:100 and secondary antibody DAR Alexa Fluor™ Plus 488 (cat. no. A32790, Thermo Fisher Scientific) was diluted 1:2000. (B) Negative control image without primary MAS1 antibody. Immunofluorescence was conducted according to Method 3 "immunofluorescence" of the main manuscript.
