# Supplementary material for: Upregulation of angiotensin-(1–7) formation in human podocytes – enzyme activity assay upon fluid flow shear stress
Source: PLoS One. 2026 Jan 9;21(1):e0339874. doi: 10.1371/journal.pone.0339874 (PMC12788633; doi:10.1371/journal.pone.0339874)
Supplement: S3 Data — (PDF) [file pone.0339874.s011.pdf]

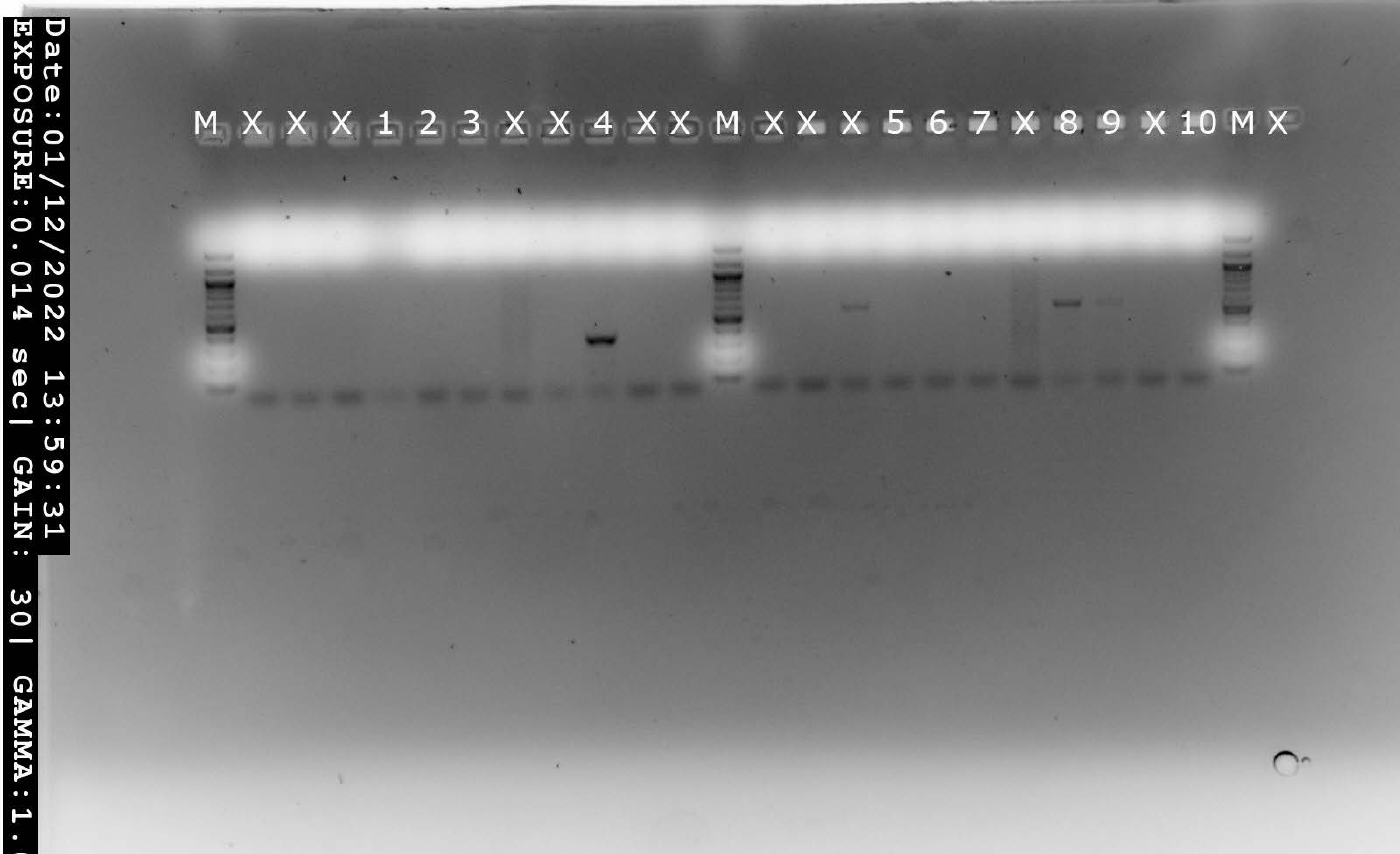

S1Fig lane 1-4 panel A, lane 5-10 panel B
M: 100 bp DNA ladder marker (cat. no. N3231L, New England BioLabs, Ipswich, MA, US), lane 1: hPC sample, lane 2: hPC sample, lane 3: hPC sample, lane 4: human liver sample, lane 5: hPC sample, lane 6: hPC sample, lane 7: hPC sample, lane 8: huan lung sample, lane 9: human liver sample, lane 10: water

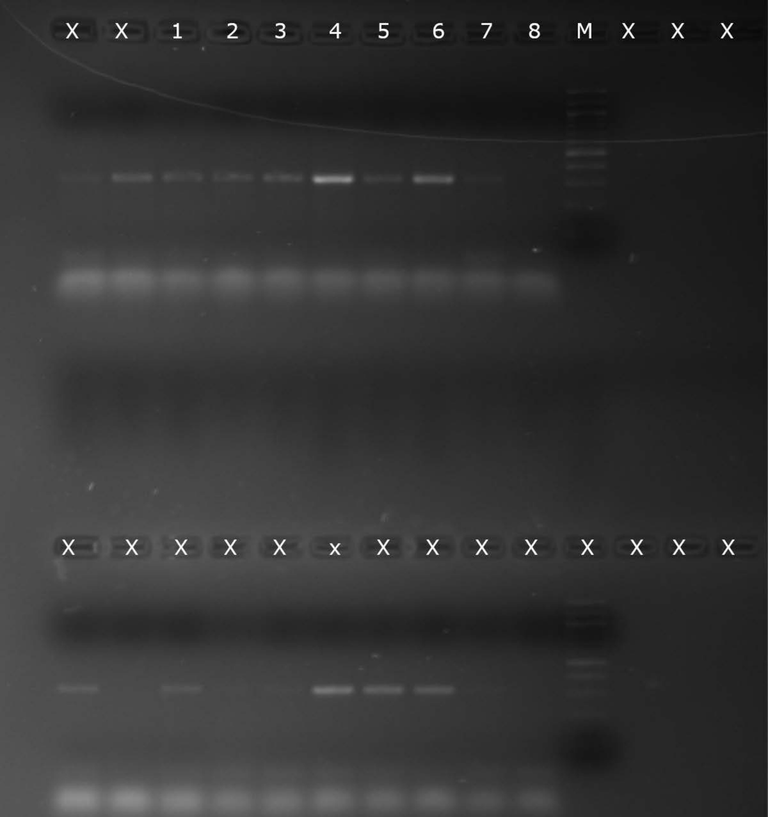

S1 Fig, panel C
lane 1: hPC sample, lane 2: hPC sample, lane 3: hPC sample,
lane 4: human lunge smaple, lane 5: human lunge sample,
lane 6: human testis sample, lane 7: human testos sample, lane 8: water, M: 100 bp DNA ladder marker (cat. no. N3231L, New England Biolabs, Ipswich, Ma, US)

Date:10/11/2023 14:32:50
EXPOSURE:0.010 sec| GAIN: 0| GAMMA:1.0

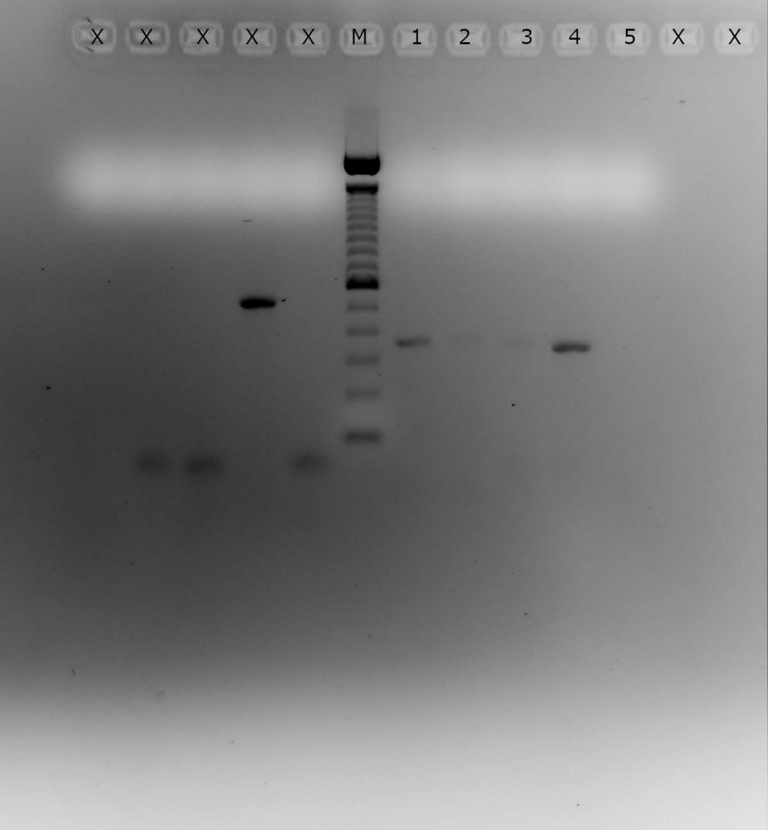

S1 Fig, panel D
M: 100 bp DNA ladder marker (cat. no. N3231L, New England BioLabs, Ipswich, MA, US), lane 1: hPC sample, lane 2: hPC sample, lane 3: hPC sample, lane 4: human testis sample, lane 5: water

Date:08/09/2020 12:38:32
EXPOSURE:0.024 sec| GAIN: 0| GAMMA:1.0

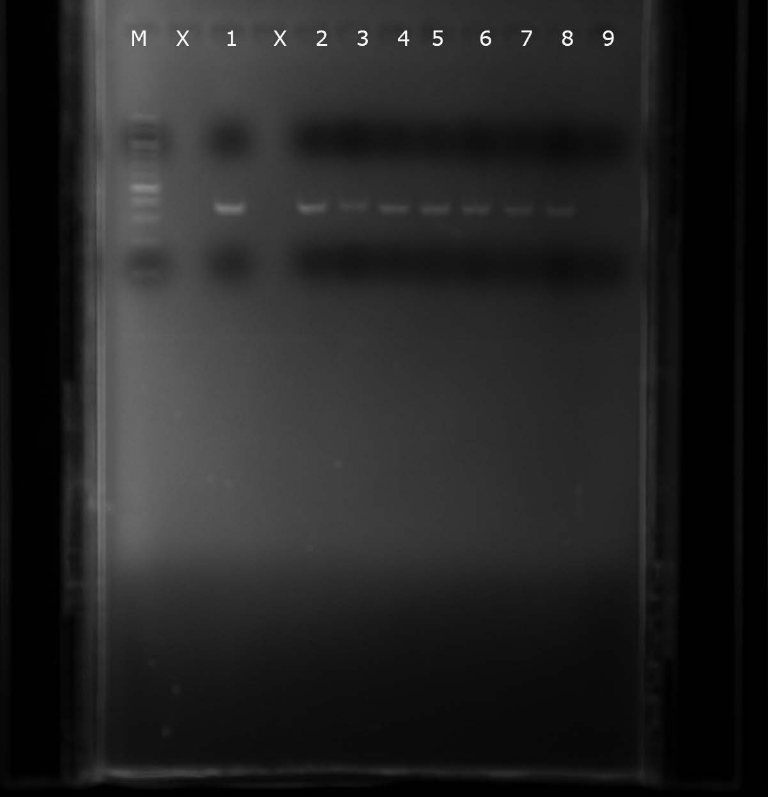

S1 Fig, panel E
M: 100 bp DNA ladder marker (cat. no. N3231L, New England BioLabs, Ipswich, MA, US), lane 1: hPC sample, lane 2: hPC sample, lane 3: human placenta sample, lane 4: human placenta sample, lane 5: human testis sample, lane 6: human testis sample, lane 7: human liver sample, lane 8: human liver sample, lane 9: water

Date:21/07/2022 13:30:39
EXPOSURE:0.014 sec| GAIN: 0| GAMMA:1.0

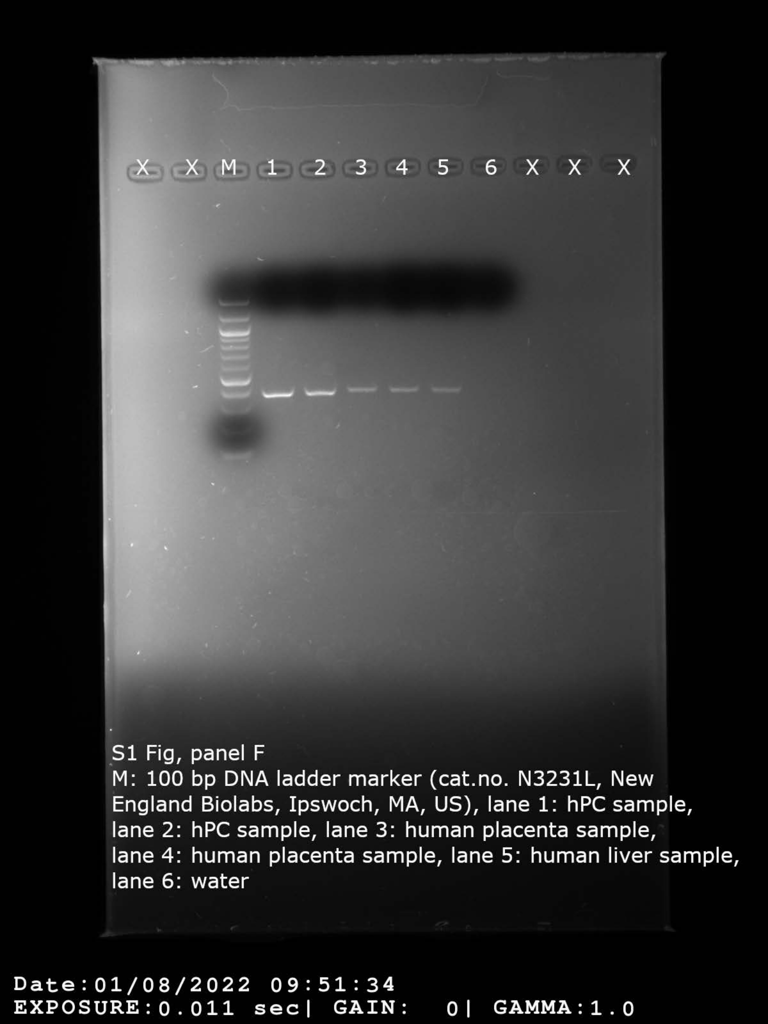

S1 Fig, panel F
M: 100 bp DNA ladder marker (cat.no. N3231L, New England Biolabs, Ipswoch, MA, US), lane 1: hPC sample, lane 2: hPC sample, lane 3: human placenta sample, lane 4: human placenta sample, lane 5: human liver sample, lane 6: water

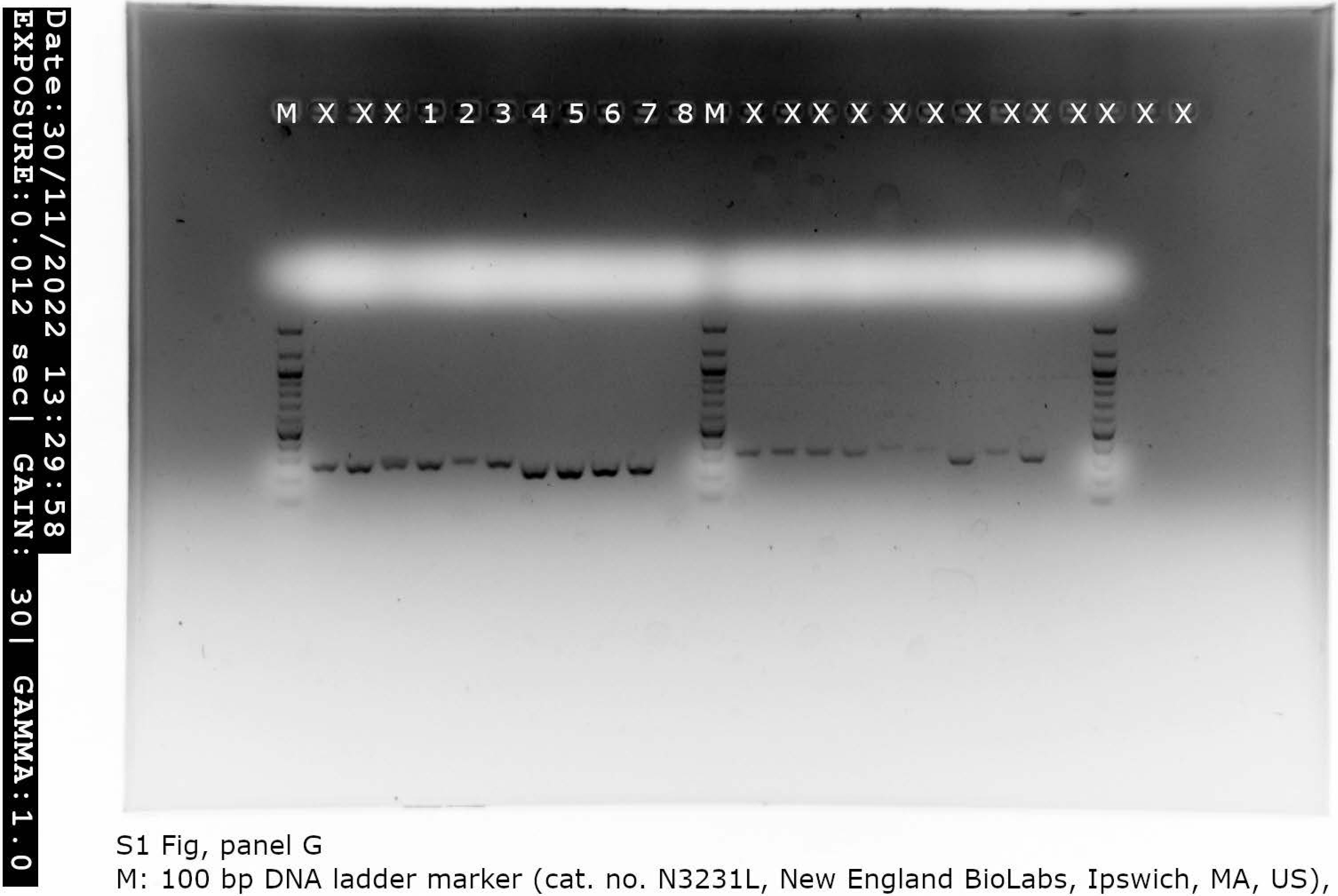

S1 Fig, panel G
M: 100 bp DNA ladder marker (cat. no. N3231L, New England BioLabs, Ipswich, MA, US), lane 1: hPC sample, lane 2: hPC sample, lane 3: hPC sample, lane 4: human lung sample, lane 5: human placenta sample, lane 6: human liver sample, lane 7: human testis sample, lane 8: water

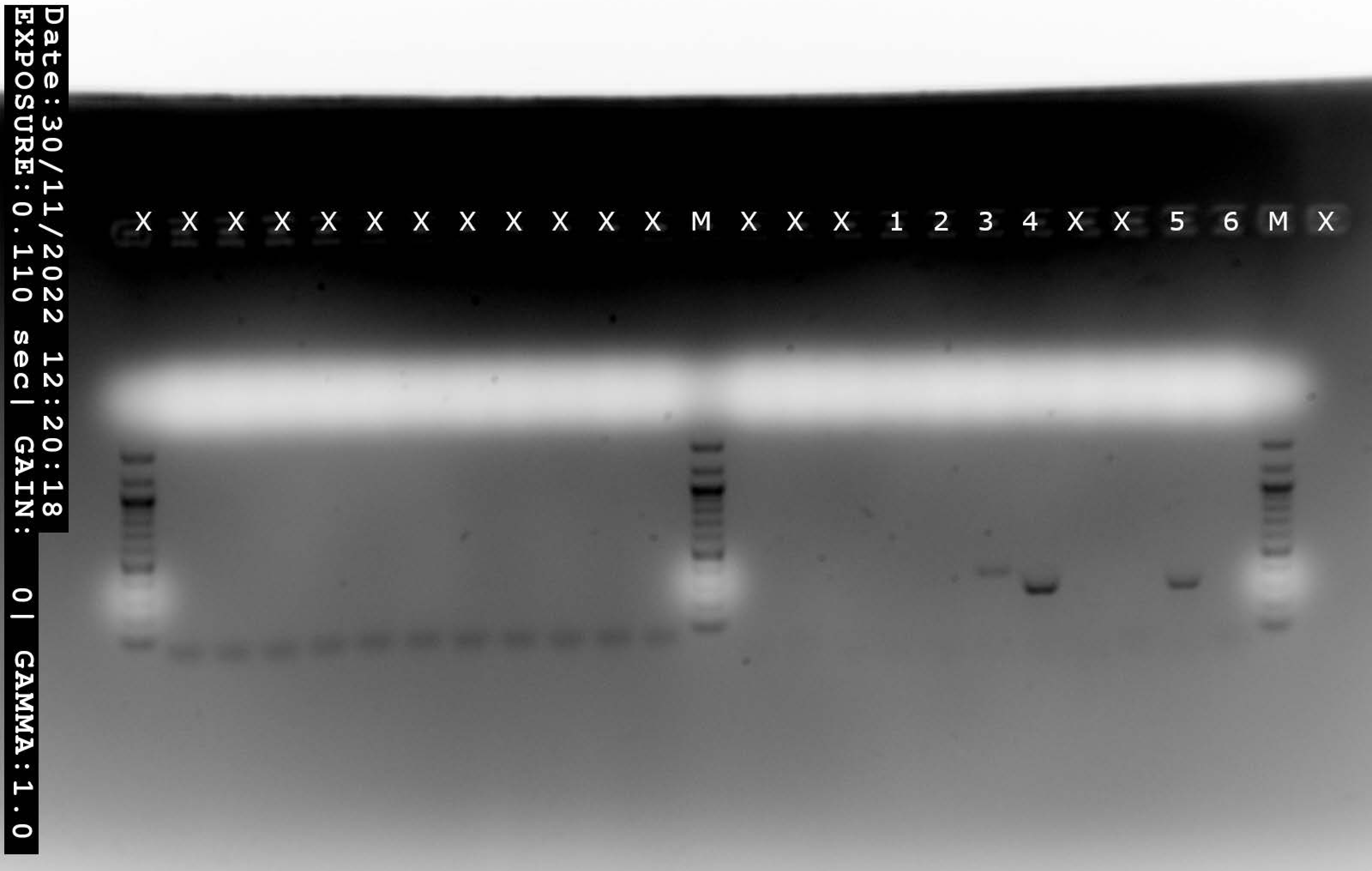

S1 Fig, panel H

M:100 bp DNA ladder marker (cat. no. N3231L, New England BioLabs, Ipswich, MA, US), lane 1: hPC sample, lane 2: hPC sample, lane 3: hPC sample, lane 4: human lung sample, lane 5: human testis sample, lane 6: water

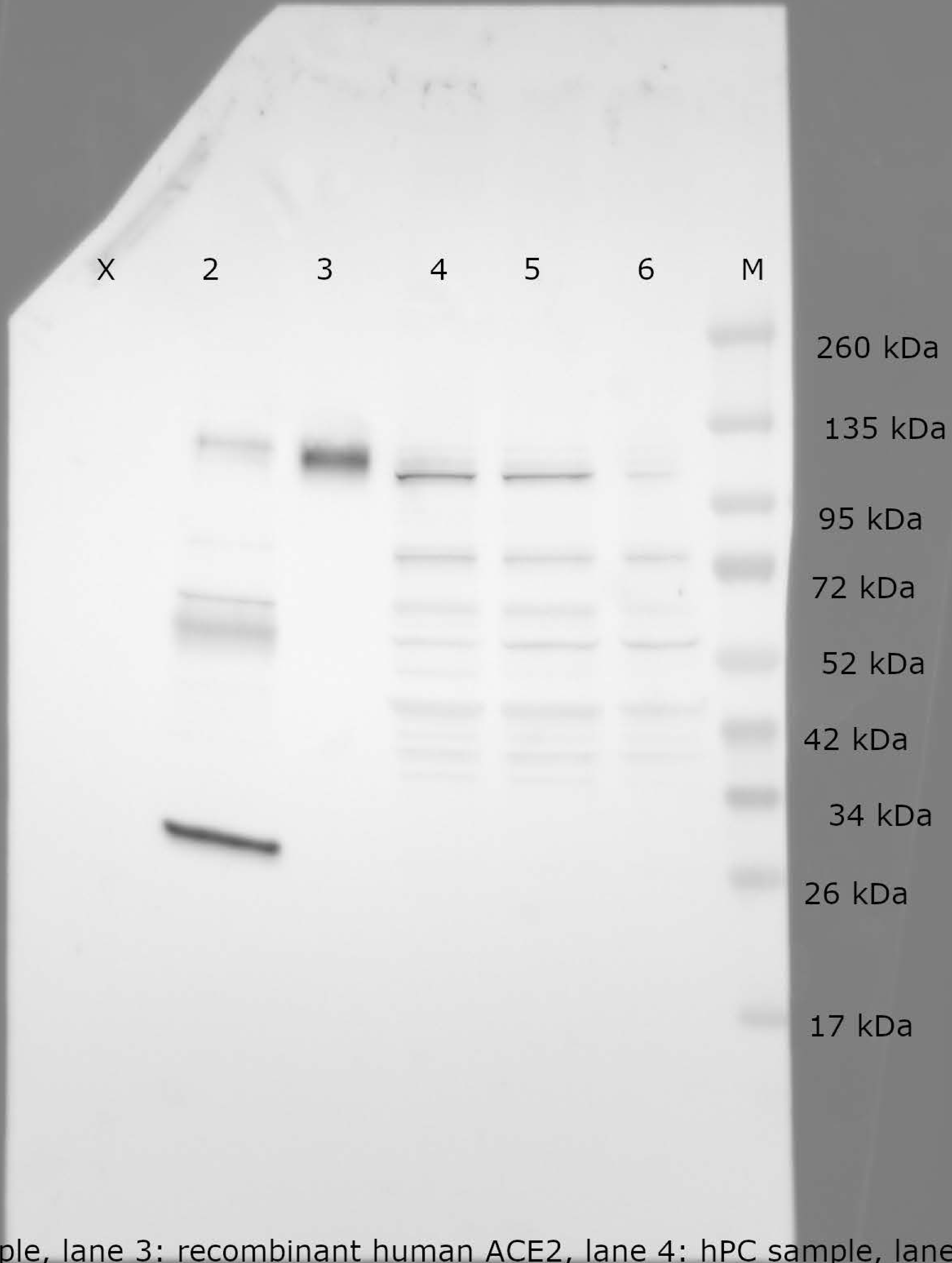

S4 Fig, panel A ACE2
lane 2: human kidney sample, lane 3: recombinant human ACE2, lane 4: hPC sample, lane 5: hPC sample, lane 6: hPC sample, M: Marker (Multicolor Broad Range Protein Ladder, Thermo Fisher Scientific, cat. no. #26634), chemiluminescence was measured with the Fusion Fx7 (peqlab Biotechnologie GmbH, Erlangen, Germany), exposure time 30 sec

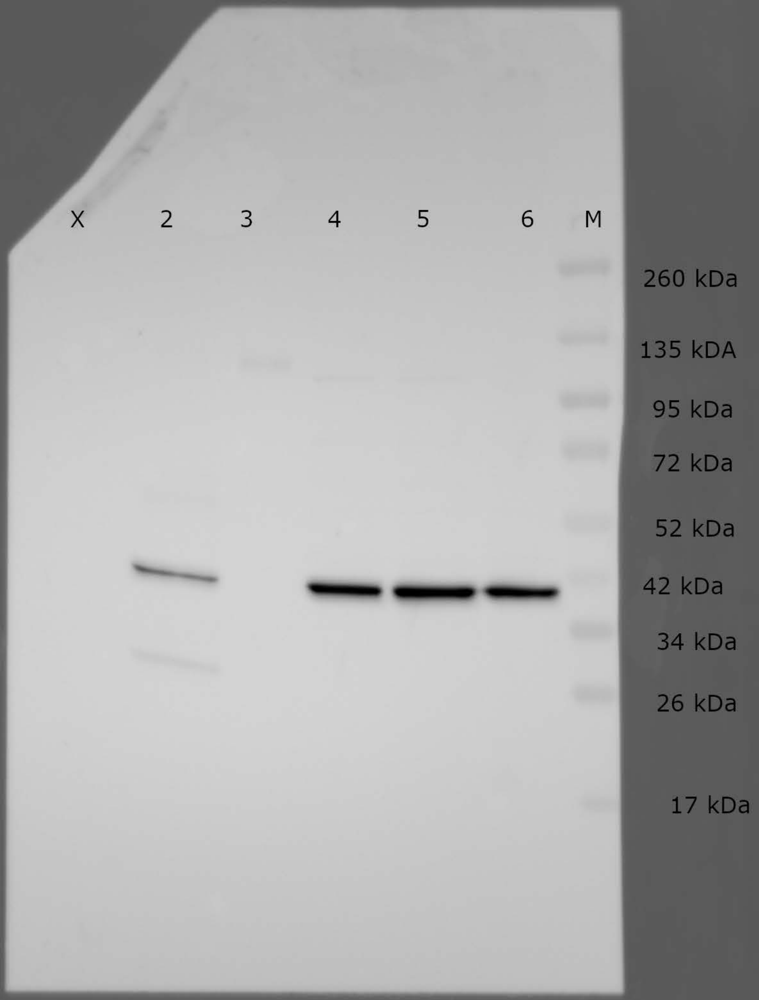

S4 Fig, panel A GAPDH
lane 2: human kidney sample, lane 3: recombinant human ACE2, lane 4: hPC sample, lane 5: hPC sample, lane 6: hPC sample, M:Marker (Multicolor Broad Range Protein Ladder, Thermo Fisher Scientific, cat.no.#26634), chemiluminescence was measured with the Fusion Fx7 (peqlab Biotechnologie GmbH, Erlangen, Germany), exposure time 2min

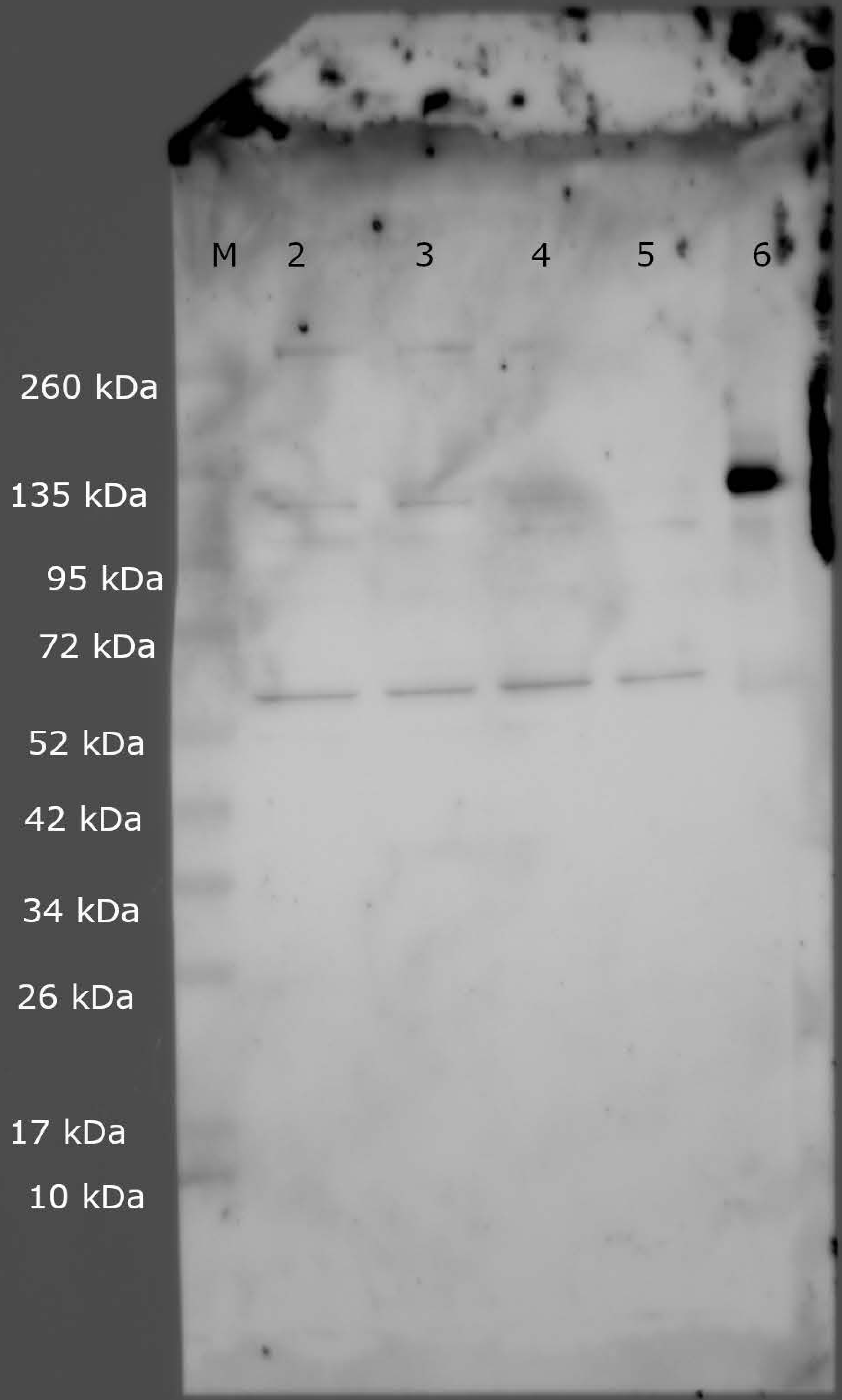

S4 Fig, panel B ACE2
M: Marker (Multicolor Broad Range Protein Ladder, Thermo Fisher Scientific, cat. no. #26634), lane 2: hPC sample, lane 3: hPC sample, lane 4: hPC sample, lane 5: hPC sample, lane 6: human kidney sample, chemiluminescence was measured with the Fusion Fx7 (peqlab Biotechnologie GmbH, Erlangen, Germany) exposure time 2 min

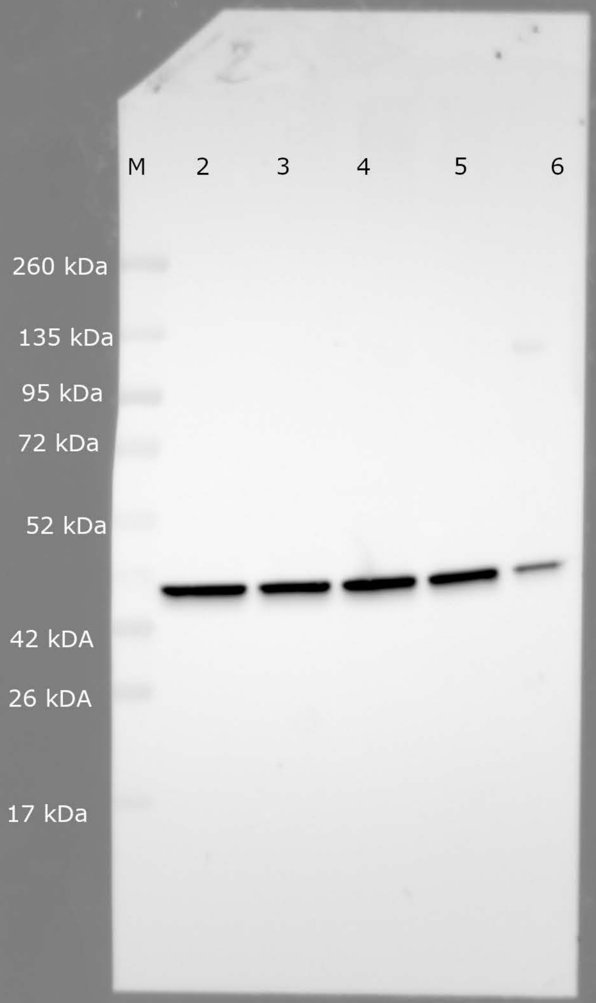

S4 Fig, panel B GAPDH
M: Marker (Multicolor Broad Range Protein Ladder, Thermo Fisher Scientific, cat. no. #26634), lane 2: hPC sample, lane 3: hPC sample, lane 4: hPC sample, lane 5: hPC sample, lane 6: human kidney sample,
chemiluminescence was measured with the Fusion Fx7 (peqlab Biotechnologie GmbH, Erlangen, Germany),exposure time 29 sec

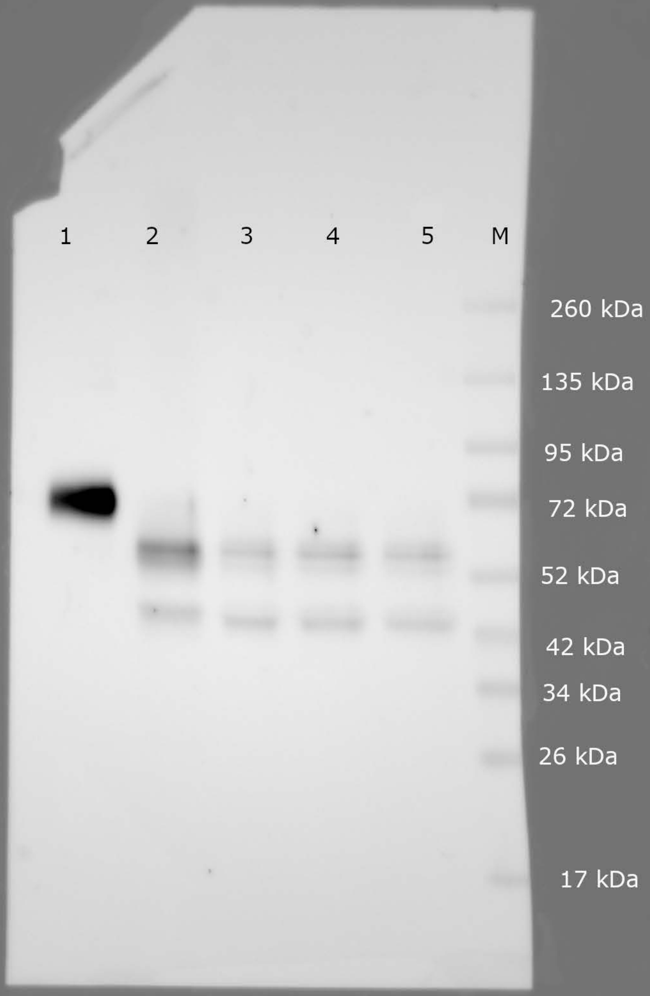

S4 Fig, panel C PRCP

lane 1: recombinant human PRCP, lane 2: human kidney sample, lane 3: hPC sample, lane 4: hPC sample, lane 5: hPC sample, M: Marker (Multicolor Broad Range Protein Ladder, Thermo Fisher Scientific, cat. no. #26634), chemiluminescence was measured with the Fusion Fx7 (peqlab Biotechnologie GmbH, Erlangen, Germany), exposure time 8 min

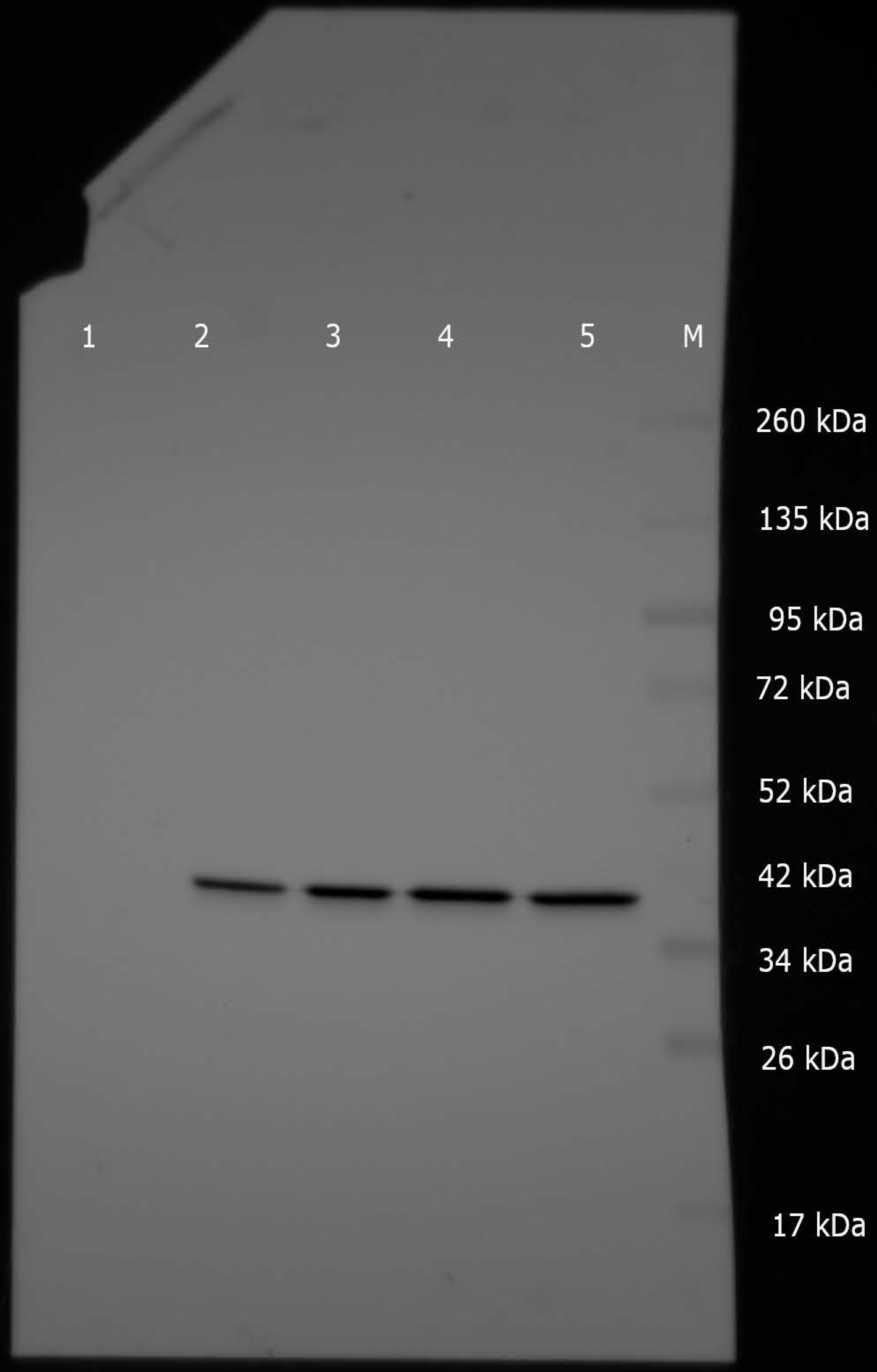

S4 Fig, panel C GAPDH
lane 1: recombinant human PRCP, lane 2: human kidney sample, lane 3: hPC sample, lane 4: hPC sample, lane 5: hPC sample, M: Marker (Multicolor Broad Range Protein Ladder, Thermo Fisher Scientific, cat. no. #26634), chemiluminescence was measured with the Fusion Fx7 (peqlab Biotechnology GmbH, Erlangen, Germany), exposure time 1 min
