# Supplementary material for: Upregulation of angiotensin-(1–7) formation in human podocytes – enzyme activity assay upon fluid flow shear stress
Source: PLoS One. 2026 Jan 9;21(1):e0339874. doi: 10.1371/journal.pone.0339874 (PMC12788633; doi:10.1371/journal.pone.0339874)
Supplement: Attachment — Submitted filename: Responses to Reviewers.docx [file pone.0339874.s013.docx]

**PONE-D-25-20067 - Revision**

**Response to Reviewers and Editors**

**Response to Editors**

We thank the Editor for the careful evaluation of our manuscript. Your valuable comments have helped us to substantially improve the clarity and precision of our work.

As a general note, all page and line numbers in our responses refer to the revised and marked manuscript version titled *“Revised Article with Changes Highlighted”*, unless otherwise specified.

1.Please ensure that your manuscript meets PLOS ONE's style requirements, including those for file naming. The PLOS ONE style templates can be found at

https://journals.plos.org/plosone/s/file?id=wjVg/PLOSOne_formatting_sample_main_body.pdf and

<https://journals.plos.org/plosone/s/file?id=ba62/PLOSOne_formatting_sample_title_authors_affiliations.pdf>

Response: We carefully checked the PLOS ONE’s style requirements and adjusted several headings to sentence case:

- Page 5, line 108: Cell culture
- Page 5, line 122: RNA isolation and reverse transcription
- Page 10, line 213: Quantitative real-time PCR

Additionally, we deleted the numbers from the subheadings of the Method section and we adjusted the naming of our Supporting Information files at the end of the revised manuscript in the section titled “Supporting Information” (page 42, line 955-975).

2. Thank you for stating the following in the Competing Interests section:

“I have read the journal's policy and the authors of this manuscript have the following competing interests: Oliver Domenig and Marko Poglitsch had a paid employment at Attoquant Diagnostics GmbH within the last 5 years.”

We note that one or more of the authors are employed by a commercial company: Attoquant Diagnostics GmbH

1. Please provide an amended Funding Statement declaring this commercial affiliation, as well as a statement regarding the Role of Funders in your study. If the funding organization did not play a role in the study design, data collection and analysis, decision to publish, or preparation of the manuscript and only provided financial support in the form of authors' salaries and/or research materials, please review your statements relating to the author contributions, and ensure you have specifically and accurately indicated the role(s) that these authors had in your study. You can update author roles in the Author Contributions section of the online submission form.

Please also include the following statement within your amended Funding Statement.

“The funder provided support in the form of salaries for authors [insert relevant initials], but did not have any additional role in the study design, data collection and analysis, decision to publish, or preparation of the manuscript. The specific roles of these authors are articulated in the ‘author contributions’ section.”

If your commercial affiliation did play a role in your study, please state and explain this role within your updated Funding Statement.

Response: As requested, our amended Funding Statement now reads:

“Oliver Domenig and Marko Poglitsch had a paid employment at Attoquant Diagnostics GmbH within the last 5 years. Attoquant Diagnostics GmbH provided support in the form of salaries for authors [OD, MP], but did not have any additional role in the study design, data collection and analysis, decision to publish, or preparation of the manuscript. The specific roles of these authors are articulated in the Author Contributions section.”

We reviewed our statements relating to the author contributions, and indicated the roles that these authors had in our study correctly. Thus, an update in the Author Contributions section of the online submission form is not necessary.

2. Please also provide an updated Competing Interests Statement declaring this commercial affiliation along with any other relevant declarations relating to employment, consultancy, patents, products in development, or marketed products, etc.

Within your Competing Interests Statement, please confirm that this commercial affiliation does not alter your adherence to all PLOS ONE policies on sharing data and materials by including the following statement: "This does not alter our adherence to  PLOS ONE policies on sharing data and materials.” (as detailed online in our guide for authors http://journals.plos.org/plosone/s/competing-interests) . If this adherence statement is not accurate and  there are restrictions on sharing of data and/or materials, please state these. Please note that we cannot proceed with consideration of your article until this information has been declared.

Please include both an updated Funding Statement and Competing Interests Statement in your cover letter. We will change the online submission form on your behalf.

Response: As requested, our updated Competing Interests Statement now reads:

“Oliver Domenig and Marko Poglitsch had a paid employment at Attoquant Diagnostics GmbH within the last 5 years. This commercial affiliation does not alter our adherence to PLOS ONE policies on sharing data and materials.”

We included both the updated Funding Statement and Competing Interests Statement in our cover letter.

3. When completing the data availability statement of the submission form, you indicated that you will make your data available on acceptance. We strongly recommend all authors decide on a data sharing plan before acceptance, as the process can be lengthy and hold up publication timelines. Please note that, though access restrictions are acceptable now, your entire data will need to be made freely accessible if your manuscript is accepted for publication. This policy applies to all data except where public deposition would breach compliance with the protocol approved by your research ethics board. If you are unable to adhere to our open data policy, please kindly revise your statement to explain your reasoning and we will seek the editor's input on an exemption. Please be assured that, once you have provided your new statement, the assessment of your exemption will not hold up the peer review process.

Response: We acknowledge this important note by the Editors and decided to include our entire data within the Supporting Information. Excel-file S1_Data contains primary LC-MS/MS data and S2_Data Excel-file calculation of delta delta Ct values for gene expression analyses of ACE2 and PRCP.

4. We note that you have included the phrase “data not shown” in your manuscript. Unfortunately, this does not meet our data sharing requirements. PLOS does not permit references to inaccessible data. We require that authors provide all relevant data within the paper, Supporting Information files, or in an acceptable, public repository. Please add a citation to support this phrase or upload the data that corresponds with these findings to a stable repository (such as Figshare or Dryad) and provide and URLs, DOIs, or accession numbers that may be used to access these data. Or, if the data are not a core part of the research being presented in your study, we ask that you remove the phrase that refers to these data.

Response: Thank you for this comment. We added the information in the Supporting Information files as S5_Fig and S6_Fig. We updated the corresponding parts in the revised manuscript and deleted the phrase “data not shown”.

5. PLOS ONE now requires that authors provide the original uncropped and unadjusted images underlying all blot or gel results reported in a submission’s figures or Supporting Information files. This policy and the journal’s other requirements for blot/gel reporting and figure preparation are described in detail at https://journals.plos.org/plosone/s/figures#loc-blot-and-gel-reporting-requirements and https://journals.plos.org/plosone/s/figures#loc-preparing-figures-from-image-files. When you submit your revised manuscript, please ensure that your figures adhere fully to these guidelines and provide the original underlying images for all blot or gel data reported in your submission. See the following link for instructions on providing the original image data: https://journals.plos.org/plosone/s/figures#loc-original-images-for-blots-and-gels.

In your cover letter, please note whether your blot/gel image data are in Supporting Information or posted at a public data repository, provide the repository URL if relevant, and provide specific details as to which raw blot/gel images, if any, are not available. Email us at plosone@plos.org if you have any questions.

Response: We carefully checked the requirements for uncropped and unadjusted images underlaying blot and gel data as reported in the author guidelines. We have added the newly acquired western blot data to our raw data file. Raw images were annotated and compiled according to the author guidelines. Briefly, original images were annotated using GIMP and exported as tif-files. Tif-files were compiled into one pdf file using Adobe Acrobat. The blot/gel image data are in the Supporting Information “S1_raw_data” file. We added this information in our cover letter.

6. If the reviewer comments include a recommendation to cite specific previously published works, please review and evaluate these publications to determine whether they are relevant and should be cited. There is no requirement to cite these works unless the editor has indicated otherwise.

Response: Reviewers did not recommend to cite specific previously published works.

**Response to Reviewer 1 Comments**

Comment 1.1

In this manuscript, authors investigated the conversion of Ang II to Ang-(1-7) in human podocytes (hPCs) both intracellularly and at the cell surface under fluid flow shear stress (FFSS). To do this, recombinant Ang II was added in hPC lysates (representing the intracellular state) or in the culture (supernatant) of the intact cells (representing the cell surface state). The converted products Ang-(1-7) were measured by means of LC-MS/MC. RT-qPCR and immunofluorescence were used to ascertain the presence of ACE2 and PRCP in hPCs. Authors concluded that more Ang-(1-7) was formed intracellularly under the FFSS treatment, but the conversion of Ang II to Ang-(1-7) stayed steady at the cell surface regardless of FFSS. With the aid of selected enzyme inhibitors, PRCP was found responsible for the Ang-(1-7) formation inside the cells while the conversion at the cell surface involved both ACE2 and PRCP enzymes. Authors also suggested that PRCP-derived Ang-(1-7) formation was enhanced upon FFSS in the supernatants of hPCs.
The rationale of using cell lysates to represent the intracellular state or the supernatant to represent the cell surface state was not explained. If cell lysates contain all intracellular proteins and cell surface proteins, and if added Ang II and the enzyme inhibitors were internalized into the intact hPCs during the one-hour incubation time, how would the result be interpreted? Where would be the major site of the conversion, inside the cells or on the cell surface?

Response:

We thank the Reviewer for the careful evaluation of our manuscript. Your valuable comments have helped us to substantially improve the clarity and precision of our work.

As a general note, all page and line numbers in our responses refer to the revised and marked manuscript version titled *“Revised Article with Changes Highlighted”*, unless otherwise specified.

In addition, all raw data are now provided in the revised Supplementary Material.

Since whole-cell lysates contain both intracellular and membrane-associated proteins, we agree that the term “intracellular” was inaccurate in this context. We have carefully revised the manuscript and removed the term “intracellular” from page 9, line 223; page 13, line 281; page 15, lines 339 and 347; and page 26, line 386 of the original version. To improve accuracy, we now explicitly state “in cell lysates” where appropriate. For example, on page 16, lines 379–381, the text now reads:

“In contrast to elevated Ang-(1-7) formation in cell lysates, no effect was observed on Ang-(1-7) formation rate at the cell surface of hPC (Fig. 3B).”

Similarly, the revised text on page 19, lines 447–448, reads:
“However, Ang-(1-7) formation in hPC whole-cell lysates is mainly driven by PRCP.”

In the Discussion section (page 22, lines 502–503), we revised the sentence to:
“Our results revealed an increased conversion of Ang II to Ang-(1-7) in hPC lysates exposed to FFSS.”

For the measurements of Ang-(1–7) formation in whole-cell lysates, cells were sonicated before the addition of spiked Ang II and enzyme inhibitors. This procedure was described in the original Methods section 7.1 (page 10, lines 220–224):

“Cell suspensions were sonicated for complete dissolution using Vibra-Cell™ (Sonics & Materials Inc., Newtown, CT, USA) and normalized to total protein concentration determined using the BIO-RAD Protein Assay Dye with bovine serum albumin as protein standard (cat. no. 5000006, Hercules, CA, USA).”

Therefore, internalization of Ang II is not relevant in this context.

In contrast, for extracellular Ang-(1–7) formation in supernatants, Ang II and enzyme inhibitors were added to intact cells. During the one-hour incubation period, receptor-mediated internalization of Ang II could have occurred. However, such potential internalization would have affected both experimental conditions (control and FFSS) equally. Importantly, Ang II was added in saturating concentrations, and residual substrate levels were measured after incubation to confirm that enzyme activities were not substrate-limited. In addition, there is, to our knowledge, no rationale for enzyme inhibitors to undergo internalization in intact cells.

Regarding the Reviewer’s question about the major site of Ang II conversion to Ang-(1-7), we consider it most likely to occur inside the cells. This is supported by the low contribution of ACE2 to Ang-(1-7) formation in whole-cell lysates and the presumably higher intracellular content of PRCP. A direct quantitative comparison between lysates and supernatants is not feasible, however, since Ang-(1-7) formation in lysates was normalized to protein content, whereas absolute values were determined in supernatants.

Comment 1.2
The presentation and interpretation of the data were somewhat convoluted and hard to follow.

Response: We thank the reviewer for this overall comment. We acknowledge, that the flow of the manuscript may have been difficult to follow for the reader. To improve clarity and readability, we thoroughly revised the manuscript and expanded on the main findings and their implications within the Results section (see also Reviewer 2, comment 6).

To enhance data presentation and to facilitate interpretation, we have incorporated Supplementary Figure S2A-D into the main text of the revised manuscript, now presented as Figure 2, in line with the recommendation of Reviewer 2.

In addition, we restructured the manuscript to provide a more logical sequence of methods and results. Specifically, the methodology and results of the inhibitor testing were moved forward to directly follow enzyme characterization. This section now appears as Methods section 4 (“Inhibition of Ang-(1-7) formation by different inhibitors,” page 7, line 163 ff.) and in the revised Results section as “Enzymes involved in Ang-(1-7) formation from substrate Ang II” (page 15, line 339 ff.).

Finally, the presentation of the data in the original Figures 4 and 5 (now revised Figures 5 and 6) has been improved for greater clarity. For further details on these revisions, please see our response to Comment 1.12.

Comment 1.3

Authors reported that the PRCP mRNA expression in hPCs was slightly decreased upon FFSS, yet the conversion of Ang II to Ang-(1-7) was increased intracellularly (cell lysate) under FFSS. This conversion was mainly driven by PRCP in cell lysates and enhanced at the cell surface (in supernatant) under FFSS. Please comment.

Response:

We thank the Reviewer for this insightful comment. In our measurements of PRCP mRNA expression in cell lysates, we observed a slight decrease under FFSS (revised Figure 4). In contrast, the total Ang-(1–7) formation rate was significantly increased under FFSS conditions (revised Figure 3). As the Reviewer correctly notes, Ang-(1–7) formation in cell lysates is mainly mediated by PRCP (revised Figure 5).
Although PRCP mRNA levels were reduced, we consider it unlikely that this two-hour stimulus is sufficient to affect protein abundance. Instead, the observed increase in enzymatic activity is more plausibly explained by post-translational modifications. Importantly, we did not detect any change in the relative contribution of PRCP to Ang-(1–7) formation between control and FFSS conditions in cell lysates (revised Figure 5). These findings suggest that, in addition to PRCP, another enzyme may contribute to the enhanced Ang-(1–7) formation observed under FFSS in cell lysates.
As we already noted in the original manuscript (page 15, lines 345–347): “Despite dual ACE2 and PRCP inhibition, residual Ang-(1-7) formation remained, which was even higher under FFSS conditions compared to controls.” This interpretation is further supported by the Discussion (original manuscript, page 21, lines 457–461), where we state:
“The remaining Ang-(1-7) formation upon dual ACE2 and PRCP inhibition is suggestive of an additional enzyme involved in the conversion of Ang II in hPC lysates contributing to the increased Ang-(1-7) production observed upon FFSS. Nevertheless, Ang-(1-7) formation in hPC lysates was predominantly driven by PRCP.”

In supernatants, following FFSS, our data indicate that PRCP-derived Ang-(1–7) formation was slightly enhanced (Figure 5B / revised Figure 6B). Several explanations may account for this observation. First, PRCP activity could be increased through post-translational modifications. Alternatively, FFSS might facilitate the transport of PRCP to the cell surface or into the supernatant via exocytosis, thereby increasing extracellular enzyme activity. Supporting this interpretation, PRCP has previously been reported to be highly concentrated in lysosomes and to retain activity when released extracellularly, either in soluble form or as part of a membrane-bound complex [1]. This possibility was already noted in the Discussion of the original manuscript (pages 20–21, lines 452–454): “This might result from increased enzyme transport to the cell surface through exocytosis or increased enzyme activity due to post-translational modification.”

Comment 1.4
As authors indicated in the manuscript, Ang II was added into each of the testing samples as the substrate for its conversion to Ang-(1-7). Why would this in vitro data be extrapolatable to the in vivo setting of glomerular hyperfiltration?

We thank the Reviewer for this valuable comment. FFSS stimulation has been reported to induce cellular responses and morphological changes similar to those observed in podocytes under glomerular hyperfiltration [2-5]. Accordingly, FFSS is widely accepted as an in vitro model to mimic the effects of glomerular hyperfiltration on podocytes. Within this framework, our study aimed to evaluate the enzymatic activity of ACE2 and PRCP in converting Ang II to Ang-(1-7).

We acknowledge that the concentration of Ang II used in our experiments (1 µg/mL) does not reflect in vivo peptide levels. However, we deliberately selected this concentration to provide optimal assay conditions. By applying Ang II in saturating amounts, we ensured that substrate availability remained in excess throughout the one-hour incubation period. This approach prevented artificial limitations on enzyme activity and allowed us to assess the catalytic capacity of ACE2 and PRCP under standardized and reproducible conditions.

We acknowledge this important limitation and had already addressed it in the Discussion of the original manuscript (page 30, lines 490–494):
“As a limitation of our study, besides the in vitro setting of cell culture, we had to exogenously add Ang II, as the endogenous concentrations were too low to detect the cleavage pattern by the enzymes investigated. However, this approach enabled us to use spiked Ang II as a substrate, allowing clear identification of only the spiked product via mass spectrometry and the precise calculation of enzyme activity.”

We agree that this experimental requirement represents a constraint. At the same time, the use of exogenously added Ang II provided a methodological advantage, as it ensured reliable product detection and robust quantification of enzyme activity under defined conditions.

Comments 1.5

Some specific concerns are listed below.
The data presented in the manuscript did not support the manuscript title which suggested a glomerular hyperfiltration setting.

Response:

We acknowledge that our in vitro model can only mimic aspects of the hyperfiltration state and does not fully reflect the in vivo situation of glomerular hyperfiltration. As explained in our response to Comment 1.4, applying FFSS with a shear stress device serves as an established in vitro approach to simulate the elevated ultrafiltrate flow in Bowman’s space that occurs under glomerular hyperfiltration.
To make this clearer for readers, we added a paragraph to the Introduction of the revised manuscript (page 2, lines 67–71), which now states:
“Applying FFSS on podocytes using a shear stress device represents an in vitro approach to mimic the increased ultrafiltrate flow in Bowman’s space as observed upon GH, including cellular responses such as actin cytoskeleton rearrangement and morphological alterations that contribute to podocyte injury [23–29].”

Furthermore, to address the Reviewer´s concern that our data did not align with the manuscript title, we have adapted the title accordingly. The revised title now reads:

“Upregulation of angiotensin-(1-7) formation in human podocytes - enzyme activity assay upon fluid flow shear stress”.

Comment 1.6
Figure 1. The cell integrity is poor in general. Only one cell showed somewhat co-localization. The ACE2 image appeared different from the one in the merged image. It is not convincing if ACE2 is localized in the cytosol and/or on the cell surface membrane. Expressions of ACE2, PRCP, and PREP at the protein level require confirmation by other means.

Response:

We thank the Reviewer for pointing out this mistake. As correctly noted, the green channel images in the original Figure 1 were inadvertently mixed up. We sincerely apologize for this oversight, particularly regarding the ACE2 image.

In the revised manuscript, Figure 1 has been corrected and now contains the appropriate ACE2 image, which is identical to the ACE2 merge channel (see below).

We agree with the Reviewer that cell integrity is impaired following immunofluorescence staining. Indeed, we encountered considerable difficulties in establishing a suitable fixation protocol, as fixation frequently resulted in cell loss and cell death. To minimize fixation-related stress, we tested different approaches and ultimately achieved the best preservation of cell integrity using methanol as the fixation reagent (see Methods section 3: Immunofluorescence). Nevertheless, we acknowledge that a reduction in cell integrity after fixation could not be fully avoided.
Despite this limitation, the main localization of ACE2 expression at the cell surface has been consistently demonstrated [6-9] and was specifically confirmed by Marquez et al. in mouse podocytes using immunofluorescence [10].

Furthermore, in response to Reviewer 2’s comments on Figure 1 and following our additional quantitative co-localization analyses, we have decided to omit the IF images of LAMP1 in order to enhance the clarity of the Results section. For further details, please see our responses to Reviewer 2 (Comments 4 and 5).

Fig 1. Characterization of ACE2, PRCP, and PREP on protein level in hPC conducted by immunofluorescence.

As suggested, we additionally performed western blot analyses to support evidence of protein expression in hPC besides IF. Since our inhibitor experiments indicated that only ACE2 and PRCP contribute to the conversion of Ang II to Ang-(1-7), we focused on detecting ACE2 and PRCP by western blot. The respective methodological details and results have been added to the revised Supplement. Accordingly, the revised manuscript was modified as follows:

1. In the revised Methods section page 7, lines 160-161 it now reads: “Additionally, ACE2 and PRCP were determined on protein level in hPC by western blot (Supplement Method S2).”
2. In the revised Supplement Methods S2 it now reads: “ACE2 and PRCP were additionally detected on protein level by western blot. 20µg of protein was used for hPC samples and 10µg protein for human kidney samples. 0,001 µg recombinant ACE2 (cat. no. TP720353, OriGene, Rockville, Maryland, US) was applied and 0,02 µg recombinant PRCP (cat. no. 7164-SE, R&D Systems, Minneapolis, MN, US). Protein samples were normalized to 30µl using RIPA-buffer with protease and phosphatase inhibitors and were mixed with 8µl loading buffer, denatured at 95 °C for 5 min, and stored on ice. Samples were applied on a Novex™ WedgeWell™ 4 bis 20 %, Tris-Glycin, 1,0 mm, Mini-Protein-Gel (cat. no. XP04202BOX, Thermo Fisher Scientific, Waltham, MA, US) and gel electrophoresis was conducted with a XCell SureLock Mini-Cell (cat. no. EI0001, Thermo Fisher Scientific) for 80 min at 150V. Proteins were blotted on a nitrocellulose membrane (cat. no. LC2001, Thermo Fisher Scientific). ACE2 was detected using rabbit anti-ACE2 antibody (anti-ACE2-1, cat. no. sc-20998, Santa Cruz Biotechnology, Figure S4A) diluted 1:200 and additionally with rabbit anti-ACE2 antibody (anti-ACE2-2, cat. no. PA5-85139, Thermo Fisher Scientific, Figure S4B) diluted 1:300. PRCP was detected using rabbit anti-PRCP (cat. no. ATA-HPA017065, biozol, Hamburg, Germany, Figure S4C) diluted 1:1000. Primary antibodies were incubated over night at 4°C. Following washing, blots were then incubated for 1h at room temperature with horseradish peroxidase conjugated goat anti‐rabbit (cat. no. sc‐2004, Santa Cruz Biotechnology) diluted 1:5000 as the second antibody. GAPDH was detected using primary antibody rabbit-anti GAPDH (TA346868, OriGene) diluted 1:10000 and secondary antibody horseradish peroxidase conjugated goat anti‐rabbit (cat. no. sc‐2004, Santa Cruz Biotechnology) diluted 1:5000. Staining were detected using SuperSignal™ West Dura Extended Duration Substrate (cat no. 3707, Thermo Fisher Scientific) with the Fusion Fx7 (peqlab Biotechnologie GmbH, Erlangen, Germany).”
3. Western Blots are shown in the revised Supplement Figure S4A-C:

Fig S4: Western blot analyses of (A, B) ACE2 and (C) PRCP. (A) Using anti-ACE2-1, glycosylated full-length ACE2 (120 kDa) and a short ACE2 isoform (52 kDa) was detected in human kidney (10µg, positive control) and confirmed in hPC samples (20 µg) as well. Recombinant human ACE2 was detected at 120 kDa. (B) Using anti-ACE2-2, glycosylated full-length ACE2 (120 kDa) was clearly detected in human kidney and weak signals were detected in hPC samples. Short-ACE2 isoform (52 kDa) was detected in hPC samples. In contrast, human kidney only showed a faint band. (C) Human kidney as well as hPC samples revealed the same PRCP pattern (55kDa and 45 kDA). Recombinant human PRCP was detected at 70 kDa, as described by the manufacturer. (A-C) GAPDH (37kDa) detection served as loading control for human kidney and hPC samples.

4. The revised Results section on page 14 lines 322-323 now includes: “Additional western blot analyses confirmed ACE2 and PRCP expression on protein level in hPC (Fig S4A-C).”

Comment 1.7
Figure 2. The amount of Ang-(1-7) generated and detected was low in general throughout the entire study, given the amount of pure Ang II substrate added in the assays. How does this quantity relate to the physiological level of Ang-(1-7) in vivo?

Response: It is correct that the substrate concentrations used in the enzyme assays were much higher than the concentration of the product. Since our experiments were designed as enzyme activity assays, we applied spiked Ang II at saturating levels to ensure proper assay functionality without substrate limitation. Thus, the peptide concentrations used do not reflect physiological in vivo levels, as we also pointed out in our response to comment 1.4. This limitation had already been discussed in the original version of the manuscript (page 30, lines 490–494): “As a limitation of our study, besides the in vitro setting of cell culture, we had to exogenously add Ang II, as the endogenous concentrations were too low to detect the cleavage pattern by the enzymes investigated. However, this approach enabled us to use spiked Ang II as a substrate, allowing clear identification of only the spiked product via mass spectrometry and the precise calculation of enzyme activity.”

To the best of our knowledge, no validated quantitative data on Ang II and Ang1-7 levels within the human kidney has been published. Lin at al. reviewed and highlighted existing literature concerning angiotensin levels within the kidney [11]. It is well established that Ang II is the predominant angiotensin metabolite in the kidney with tissue levels reported to be 10- to 20-fold higher than corresponding plasma levels [11]. In healthy individuals, plasma Ang II levels are in the low picomolar range, approximately 5-20 pmol/L [12, 13]. Plasma Ang1-7 levels of healthy males are about 1.0 +/- 0.2 fmol/mL plasma [14].

When comparing these human plasma Ang-(1-7) levels (fmol/mL) to the Ang-(1-7) concentrations measured in supernatants (nmol/L/h) from our experiments (Figure 6, revised manuscript), our values exceed physiological plasma levels by approximately three orders of magnitude. However, this comparison should be interpreted with caution, as plasma levels represent single-point measurements, whereas our data represent enzymatic conversion rates over a one-hour incubation period. Nevertheless, we acknowledge that the substrate concentrations applied in our experimental setting were unphysiologically high.

Given that Ang II exhibits similar receptor affinity across species, it is reasonable to assume that peptide levels may also be comparable among species [11]. Multiple studies have reported rat renal Ang II concentrations on the order of several hundred fmol/g kidney tissue, with renal Ang-(1-7) levels approximately 5–10 times lower than Ang II levels [15-17], reviewed in [11]. By extrapolation human renal Ang-(1-7) concentrations may be estimated at around 50 fmol/g kidney. These values differ from our experimentally derived Ang-(1-7) formation rates (fmol/µg/h) by approximately seven orders of magnitude. As emphasized above, our measurements reflect enzymatic formation rates during a one-hour incubation period under non-physiological Ang II substrate concentrations.

Comment 1.8

The number of hPCs, the amount of cell lysates, or the volume of supernatants used for each experiment was not clearly stated. This hampered the data interpretation.

Response: Regarding the number of hPCs used for the experiments, we stated in our original manuscript in the Methods section 3 on page 14 lines 127-128 for immunofluorescence “Cells were seeded at 25 x 10³ cells per well in 8-chamber NUNC Lab-Tek II Chamber slides” and in section 4 for the FFSS experiments on page 15 lines 157-158 “3.5 × 10^5^ cells were seeded on collagen IV coated Culture Slips®”.

For the cell lysates, the total protein concentration was determined and normalized to 200µg total protein/mL before further use. In the Method section 7 of the original manuscript lines 220-226 it read:” Cell suspensions were sonicated for complete dissolution using Vibra-Cell™ (Sonics & Materials Inc., Newtown, CT; US) and normalized to total protein concentration determined using BIO-RAD Protein Assay Dye with bovine serum albumin as protein standard (cat. no. 5000006, Hercules, CA, US). Samples normalized to 200 μg total protein/mL were diluted in inhibitor solution containing Ang II as substrate (Methods 6, see above) +/- 226 10 μmol/L ACE2i and +/- 10 μmol/L PRCPi and incubated for 1 h at 37 °.”

In the revised Method section 7.1 it now reads on page 11 lines 260-262: “Samples normalized to 200 µg total protein/mL were diluted in 100µL inhibitor solution containing Ang II as substrate (Methods 4, see above) +/- 10 µmol/L ACE2i and +/- 10 µmol/L PRCPi and incubated for 1 h at 37 °.”

Regarding the volume of cell supernatants used, we specified the volume in the revised manuscript in Methods section 7.2 (lines 277–279):”100µL inhibitor solutions were applied into one chamber of the cassette onto hPC and incubated for 1 h at 37 °C.”

Comment 1.9

In some essays, the Ang-(1-7) formation rate was reported and in others the Ang-(1-7) formation was reported. What does the “rate” mean in this context? Please comment.

Response: The Ang-(1-7) formation rate describes the amount of Ang-(1-7) formed over the one-hour incubation period. This time-dependent formation applies to both cell lysates and supernatant assays. Thus, we appreciate the Reviewer´s comment and conclude that the Ang-(1-7) formation rate should be reported for both assay types. Accordingly, we adjusted wording and units in the revised manuscript to better represent the supernatant assay data. It now reads:

1. In the Method section 7.2 of the revised manuscript it now reads on page 11 lines 271-272: “Ang-(1-7) formation rate on hPC surface was determined in presence and absence of ACE2i or PRCPi after applying FFSS.” And on page 13 line 298-299:” Ang-(1-7) formation rate for cell lysates is expressed as (fmol / µg protein) / h and for supernatants as (nmol / L) / h.”
2. In the Result section of the revised manuscript it now reads on page 16 line 379-381:” In contrast to elevated Ang-(1-7) formation in cell lysates, no effect was observed on Ang-(1-7) formation rate at the cell surface of hPC (Fig 3B). “
3. In the Figure legend of revised Figure 3 it now reads in the revised manuscript on page 16 lines 387-389:” Ang-(1-7) formation rate in supernatants was not significantly regulated upon FFSS (blue squares) compared to no flow controls (black circles).”

The Figure legend headline for revised Figure 6 on page 21 line 481 now reads: “Ang-(1-7) formation rate from Ang II in hPC supernatants upon FFSS.”

4. In revised Figure 3B and in revised Figure 6A and 6B we changed the name of the y-axis into “Ang-(1-7) formation rate [(nmol/L)/h]”.

**Fig 3. Ang-(1-7) formation in hPC lysates and supernatants upon FFSS.**

Comment 1.10

Methods of LC-MS/MS were not described in detail.

Response: Methods section 7 has been updated to improve clarity, including subdivision into more descriptive subheadings (“7.1 cell lysates sample preparation”, 7.2 Cell supernatants sample preparation”, and “7.3 LC/MS-MS”).

Additionally, we incorporated further methodological details regarding the LC-MS/MS analysis in the revised Methods section 7.3 on page 12, lines 284–303: “Stable isotope-labeled internal standards for Ang II and Ang-(1-7) were added to acidified samples at 200 pg/mL. Following C18-based solid-phase extraction, samples were subjected to LC-MS analysis using a reversed-phase analytical column (Acquity UPLC C18; Waters, Milford, Massachusetts) operating in line with a XEVO TQ-S triple quadrupole mass spectrometer (Waters) in MRM mode. Internal standards were used to correct for matrix effects and peptide recovery of the sample preparation procedure for both angiotensin peptides in each individual sample. Angiotensin peptide concentrations were calculated considering the corresponding response factors determined in appropriate calibration curves, on condition that integrated signals exceeded a signal-to-noise ratio of ten. Ang II concentration was measured in a non-incubated control sample as reference and after incubation in all samples to confirm that the substrate is not exhausted following incubation. Assay controls using recombinant enzymes were included to ensure that the inhibitor concentration was functional and to exclude non-specific formation of Ang-(1-7). Ang-(1-7) formation rate for cell lysates is expressed as (fmol / µg protein) / h and for supernatants as (nmol / L) / h. In cell lysates, the sole effect of PRCP inhibition on the Ang-(1-7) formation rate was calculated by subtracting remaining Ang-(1-7) formation rate upon ACE2 inhibition in each sample from the respective total value. This value was then summed to the ACE2i + PRCPi value of the respective sample which finally represents the individual PRCPi value.”

Comment 1.11
Figure 3. The actual mRNA expression levels of ACE2 and PRCP were not reported, rather, the fold change between the control and the FFSS groups was reported, despite the neglectable difference of the expression level between sample groups.

Response: This is correct. We calculated the ΔΔCt - values and plotted the log2 fold-change (2^- ΔΔCt) a commonly used method for calculation of the relative change in expression. Ct-values are normalized to a house keeper gene and compared to control values (untreated control cells). The CT-values (NOT normalized to the house keeper gene of the sample) ranged from 27.6 to 30.5 for ACE2, and 21.2 to 23.6 for PRCP. All mRNA expression data are now included in the Supplement information of the revised manuscript.

Comment 1.12
Figure 4 and Figure 5. These were basically the same experimental design testing the formation of Ang-(1-7) in cell lysates (Figure 4) or in the supernatants (Figure 5). The combined inhibitors were used in Figure 4, but in Figure 5 single inhibitor was used. Please comment.

Response: Presenting data on combined inhibition with ACE2i + PRCPi in Figure 4 of the original manuscript originated from preliminary experiments. Thanks to your valuable comment, we realize that our original presentation of Figure 4 and 5 lacks consistency in data presentation. To facilitate reading and interpretation of the data, we therefore decided to profoundly adjust Figure 4 and Figure 5 as described below. Please note that Reviewer 2 suggested to incorporate supplementary Figure S2 (A-D) as part of the main figures. Thus, original Figure 4 has now become revised Figure 5 and original Figure 5 is now revised Figure 6.

For revised Figure 5 the following adaptions were made:

1. We now present ACE2i and PRCPi individually. We calculated the individual PRCPi effect as followed: For the ACE2 inhibitory effect, ACE2i in each sample was substracted from the respective total value. This value was then summed to the values of the ACE2i + PRCPi of the respective sample which finally represents the individual PRCPi value. This approach is possible as the specificity of the ACE2 inhibitor has been proven in advance (see revised Figure 2).
2. New statistical analyses were performed. The corresponding data are presented in revised Figure 5.
3. The name of the untreated group on the x-axis was changed from “no inhibitor” into “total” to align with revised Figure 6. Additionally, the revised Figure 5 now display the individual inhibitory effects “ACE2i” and “PRCPi”.
4. The Figure text of revised Figure 5 was changed accordingly. It now reads on page 19 lines 455-457: “Inhibition of PRCP (green triangles) significantly reduced the Ang-(1-7) formation compared to total Ang-(1-7) formation (black circles) as well as ACE2 inhibition (blue squares).”

 Fig 5. Ang-(1-7) formation rate in hPC lysates upon FFSS.

5. In the Method section of the revised manuscript the calculation of individual PRCPi effects in cell lysates was added on page 12 lines 299-303:” In cell lysates, the sole effect of PRCP inhibition on the Ang-(1-7) formation rate was calculated by subtracting remaining Ang-(1-7) formation rate upon ACE2 inhibition in each sample from the respective total value. This value was then summed to the ACE2i + PRCPi value of the respective sample which finally represents the individual PRCPi value.”
6. In the Results section of the revised manuscript we adapted the text on page 19 lines 443-447 accordingly. It now reads: “Inhibition of PRCP resulted in a significantly reduced Ang-(1-7) formation rate by at least half regardless of FFSS exposure or no flow control (p<0.0001; Fig. 5). Despite ACE2 and PRCP inhibition, a residual Ang-(1-7) formation rate remained, which was even higher under FFSS conditions compared to controls.”
7. We deleted the word “dual” in the Discussion section. It now reads in the revised version on lines 574-577: “The remaining Ang-(1-7) formation rate despite ACE2 and PRCP inhibition is suggestive of an additional enzyme involved in the conversion of Ang II in hPC lysates contributing to the increased Ang-(1-7) production observed upon FFSS.”

For revised Figure 6 the following adaptions were made:

1. The name and unit on the y-axis were changed into “Ang-(1-7) formation rate [(nmol/L)/h].
2. The data presentation was aligned with revised Figure 5. Correspondingly, the values now depict the measured Ang-(1-7) formation rate after addition of the respective inhibitor. Data thus no longer show the calculated contribution of the enzyme (originally referred to as “ACE2- or PRCP-derived”).
3. Statistical analyses were conducted with the new data.
4. The labels on the x-axis were changed into ACE2i and PRCPi.
5. The Figure text of Figure 6 of the revised manuscript was changed accordingly.

**Fig 6.** **Ang-(1-7) formation rate from Ang II in hPC supernatants upon FFSS.** (A) Under no flow control condition, Ang-(1-7) formation with ACE2 inhibition (blue squares) was significantly lower than total Ang-(1-7) formation (black circles) and formation with PRCP inhibition (green triangles). (B) Upon FFSS exposure, ACE2 inhibition (blue squares) reduced the Ang-(1-7) formation significantly compared to total Ang-(1-7) formation without inhibitors (black circles). Ang-(1-7) formation with PRCP inhibition (green triangles) was no longer significantly different to Ang-(1-7) formation upon ACE2 inhibition. Experiments were performed twice on different cell passages and different dates, each experiment consisting of n = 4 technical replicates, respectively. Statistics: **, p < 0.01; ***, p < 0.001; n.s., not significant, assessed by one-way ANOVA with Tukey`s multiple comparison follow-up test. Each data point represents one technical replicate. Data are plotted as mean ± SD (horizontal lines).

6. The Methods section was changed accordingly: The sentence in the revised manuscript on page 12 lines 280-282 was deleted:” Differences between samples with and without inhibitor were calculated to determine the respective proportion of ACE2 and PRCP contributing to the formation of Ang-(1-7).”
7. The Results section “Contribution of ACE2 and PRCP for Ang-(1-7) formation in hPCs upon FFSS”- “cell surface” was changed. In the revised manuscript it now reads on page 20 lines 470-475:” Under no flow control conditions, ACE2 inhibition (mean 3,75 nmol/L/h) reduced the Ang-(1-7) formation rate significantly compared to total Ang-(1-7) formation rate (mean 8.02 nmol/L/h) and compared to PRCP inhibition (mean 7.70 nmol/L/h) (Fig. 6A). Upon FFSS, Ang-(1-7) formation rate with PRCP inhibition (mean 5.55 nmol/L/h) was no longer significantly different from Ang-(1-7) formation rate with ACE2 inhibition (mean 3.54 nmol/L/h) (Fig 6B). This suggests, besides ACE2 being the main enzyme responsible for Ang-(1-7) formation on the cell surface, PRCP-derived Ang-(1-7) formation was enhanced upon FFSS in supernatants of hPC (Fig 6B).”

Comment 1.13
If intracellular Ang-(1-7) formation in hPCs was mainly driven by PRCP (Figure 4), following from this, would Figure 5 suggest that both ACE2 and PRCP drove the conversion of Ang II to Ang-(1-7) in the supernatant? Please comment.

Response: Yes, this is correct. Under control conditions, the Ang-(1-7) formation was mainly ACE2 driven in the supernatants. Upon FFSS, ACE2 and PRCP formed Ang-(1-7) in the supernatants. Please see the Result section of the original manuscript page 16 lines 364-368 were we stated: “Under no flow control conditions, Ang-(1-7) formation by PRCP (0.65 nmol/L) was significantly lower than formation by ACE2 (4.78 nmol/L) (Fig 5A). Upon FFSS, PRCP (2.28 nmol/L) and ACE2 (3.88 nmol/L) activity in supernatants was no longer significantly different. This suggests, that PRCP-derived Ang-(1-7) formation was enhanced upon FFSS in supernatants of hPC (Fig 5B).”

As we changed the data presentation in the revised manuscript, it now reads on page 20 lines 470-475: “Under no flow control conditions, ACE2 inhibition (mean 3.75 nmol/L/h) reduced the Ang-(1-7) formation significantly compared to total Ang-(1-7) formation (mean 8.02 nmol/L/h) and compared to PRCP inhibition (mean 7.70 nmol/L/h) (Fig. 6A). Upon FFSS, Ang-(1-7) formation with PRCP inhibition (mean 5.55 nmol/L/h) was no longer significantly different from Ang-(1-7) formation with ACE2 inhibition (mean 3.54 nmol/L/h) (Fig 6B).”

Comment 1.14

If PRCP-derived Ang-(1-7) formation was enhanced upon FFSS in supernatants, similarly, would Figure 5 suggest that ACE2-derived Ang-(1-7) formation was reduced upon FFSS in supernatant? Please comment.

Response: No, this conclusion cannot be drawn from the data. We presented the means of Ang-(1-7) formation by ACE2 in the supernatants under control conditions and upon FFSS on page 16 lines 364-367 in the Results section of the original manuscript where we stated:” Under no flow control conditions, Ang-(1-7) formation by PRCP (0.65 nmol/L) was significantly lower than formation by ACE2 (4.78 nmol/L) (Fig 5A). Upon FFSS, PRCP (2.28 nmol/L) and ACE2 (3.88 nmol/L) activity in supernatants was no longer significantly different.”

In revised Figure 6, we now present the Ang-(1-7) formation rate upon ACE2 inhibition in supernatants. Accordingly, formation does not change between control condition (mean 3.75 nmol/L/h) and FFSS (mean 3.54 nmol/L/h, revised Figure 6). In the Results section of the revised manuscript on page 20, lines 470-475 it now reads:” Under no flow control conditions, ACE2 inhibition (mean 3.75 nmol/L/h) reduced the Ang-(1-7) formation rate significantly compared to total Ang-(1-7) formation rate (mean 8.02 nmol/L/h) and compared to PRCP inhibition (mean 7.70 nmol/L/h) (Fig. 6A). Upon FFSS, Ang-(1-7) formation rate with PRCP inhibition (mean 5.55 nmol/L/h) was no longer significantly different from Ang-(1-7) formation rate with ACE2 inhibition (mean 3.54 nmol/L/h) (Fig 6B).”

Comment 1.15

448-449, “on the cell surface, ACE2 activity was found to play a more prominent role in Ang-(1-7) formation than PRCP, especially under control conditions”. If this statement is true, why would ACE2 not be a major player in Ang-(1-7) formation in cell lysates. Please clarify.

Response: As stated in the Results section of the original manuscript on page 16, lines 364-365: “Under no flow control conditions, Ang-(1-7) formation by PRCP (0.65 nmol/L) was significantly lower than formation by ACE2 (4.78 nmol/L) (Fig 5A).” Hence, since Ang-(1-7) formation rate assays in supernatants were conducted on intact hPC, ACE2 activity must have been originated from membrane-bound and/or soluble ACE2. This is in line with published data on the predominant ACE2 localization on the cell surface [6-9].

In contrast, only very low ACE2 contribution to Ang-(1-7) formation was detected in whole cell lysates (revised Figure 5). This may be attributable to a higher abundance of the PRCP enzyme, it’s primary intracellular localization and the fact that the main site of conversion is proposed to be intracellular (see response to your comment 1.1).

Comment 1.16

Overall, without clear evidence of ACE2 and PRCP expression/presence in hPCs, the results of ACE2- or PRCP-derived Ang-(1-7) formation in cell lysates or supernatants remain incidental.

Response: We respect your concerns regarding the expression and presence of ACE2 and PRCP. Nevertheless, we demonstrated RNA expression of both enzymes by RT-PCR (Supplement Figures S1 Fig D and S1Fig E) and by qPCR (Figure 3). At the protein level, ACE2 and PRCP expression in hPCs was shown using immunofluorescence, with representative images provided in Figure 1 (ACE2 and PRCP stained with GAR Alexa Fluor™ Plus488, shown in the green channel). The use of immunofluorescence to detect ACE2 has previously been validated in mouse podocytes [10, 18], and the presence of ACE2 in human podocytes has also been demonstrated by immunohistochemical analysis [19].

Additionally, we have now included western blot analyses for ACE2 and PRCP in response to this concern (see our response to comment 1.6 for details). The corresponding results are presented in the revised Supplementary. Taken together with the demonstrated specificity of the ACE2- and PRCP-selective inhibitors, we consider our data on ACE2 and PRCP expression, as well as the enzyme-specific inhibition of Ang-(1-7) formation in hPC lysates and supernatants, to provide convincing evidence.

**Response to Reviewer 2 Comments**

Comment 1

1. Fig 1- Green channels of ACE2 and PREP seem to have been duplicated.
   I think the green channel shown for ACE2 IF actually belongs to PREP IF. The authors must rectify this error.

Response:

We thank the Reviewer for the careful evaluation of our manuscript. Your valuable comments have helped us to substantially improve the clarity and precision of our work.

As a general note, all page and line numbers in our responses refer to the revised and marked manuscript version titled *“Revised Article with Changes Highlighted”*, unless otherwise specified.

In addition, all raw data are now provided in the revised Supplementary Material.

We apologize for the oversight regarding the ACE2 IF image. As correctly pointed out, the green channel images were inadvertently mixed up in the original Figure 1. In the revised Figure 1, we now provide the correct image, which is identical to the ACE2 merge channel (please see below).

Comment 2

2. Fig 1 - Figure numbers A, B and C are missing for this figure but are referenced in the text.

Response: We thank the Reviewer for this comment and added the figure numbers A, B, C, and D in the revised Figure 1 (see below).

Comment 3
3. Fig 1 - Not all cells have a colocalization between PRCP and LAMP1.

Response: This is correct and concerns regarding heterogeneity of co-localization with LAMP1 have also been raised by Reviewer 1 (Comment 1.6). Indeed, integrity of the cells following immunofluorescence staining is impaired. We faced profound difficulties establishing a suitable fixation method as fixation often resulted in cell loss and cell death. However, we did our best to minimize cell stress due to fixation, and best cell integrity was achieved by methanol as the finally chosen fixation reagent (see Methods section 3 Immunofluorescence). Still, we have to acknowledge a reduction in cell integrity after fixation. This also hampered our quantitative co-localization analyses which we carried out in response to your comment 4, please see below.

Comment 4
4. Fig 1 - It is unclear how the authors conclude that there is an apparent difference in colocalization between PREP and PRCP and LAMP1 without any quantitative measure. To me, they look similarly partially colocalized. The authors should provide quantitative measures to support their conclusion and provide some more images of different regions and replicates in the Supplementary figures to generate confidence in their data.

Response: We appreciate the comment and agree that a quantitative analysis is necessary to support our statement. As suggested by the Reviewer, we thus conducted co-localization analyses of n=6 images per enzyme as follows:

Method: To investigate the spatial co-localization of the lysosomal marker LAMP1 (channel 1, red) with the target proteins ACE2, PRCP, and PREP (channel 2, green), n=6 confocal microscopy images were analyzed using the ImageJ/Fiji software [20] with the Coloc 2 plugin. Each image was split into its respective fluorescent channels. Co-localization was assessed through multiple quantitative parameters: Pearson’s correlation coefficient (R) to determine the linear correlation between channels, Spearman’s rank correlation coefficient to evaluate monotonic relationships, and Manders’ coefficients M1 and M2 to measure the fraction of overlapping fluorescence intensities. Thresholds were determined automatically using the Costes method, which also provided a significance level (Costes P-value).

Results: Co-localization between LAMP1 and ACE2 was moderate, with a Pearson correlation coefficient averaging 0.27 ± 0.14 (above threshold). Spearman’s correlation averaged 0.59 ± 0.08. Manders’ coefficients indicated moderate overlap with tM1 = 0.75 ± 0.09 and tM2 = 0.70 ± 0.08 after thresholding. The Costes P-value was 1, indicating significance despite low coefficients.

For LAMP1 and PRCP, the co-localization was similar to that of ACE2, with Pearson’s R values of 0.24 ± 0.19 (above threshold). Spearman’s rank correlation averaged 0.55 ± 0.07. Manders’ coefficients were tM1 = 0.60 ± 0.09 and tM2 = 0.73 ± 0.11 after thresholding. The Costes P-value remained 1 for all samples.

In comparison, for LAMP1 and PREP, a strong co-localization was observed, as indicated by a mean Pearson correlation coefficient of 0.51 ± 0.07 after thresholding. The Spearman rank correlation coefficient further supported this with a mean value of 0.69 ± 0.06. Manders’ coefficients showed substantial overlap with tM1 = 0.83 ± 0.12 and tM2 = 0.82 ± 0.06 after thresholding. The Costes P-value again was consistently 1, confirming the statistical significance of the co-localization.

These findings suggest that PREP shows the strongest co-localization with the lysosomal marker LAMP1, while ACE2 and PRCP exhibit only moderate, yet statistically significant, co-localization. Although PRCP is known as a lysosomal enzyme, previous studies in HUVECs have also shown only partial co-localization of PRCP with LAMP1, which has been attributed to distinct mechanisms regulating the expression or trafficking of lysosomal components [21].

However, our analyses revealed several limitations. We analyzed the whole images and did not set a region of interest. The zero-intensity pixel were not excluded, so calculation includes pixel with no signal. This influences the statistical outcome leading to a distortion of Pearson’s and Manders’ coefficients. Thus, there is a high chance that our calculated correlations may be artificially inflated. Moreover, setting the threshold often resulted in too few pixels analyzed in the images leading to further distorted correlation values, low statistical power, and poor comparability between samples. As mentioned above in our response to comment 3, cell integrity after fixation is impaired.

Due to these limitations and given the lack of added value regarding the co-localization of the enzymes with LAMP1 (see below comment 5), we propose to omit the co-localization results from the revised manuscript.

Comment 5
5. The authors should provide the rationale why they investigated the colocalization of various enzymes and LAMP1, which will be useful for the general reader to understand this study.

Response: We appreciate the Reviewer’s helpful comment. After carefully revising the manuscript and due to the limitations stated above, we presume our data on co-localization with LAMP1 does not add reliable information to the manuscript. Therefore, we focused our presentation on our primary intention to provide evidence of ACE2, PRCP, and PREP protein expression in human podocytes. This also helps with readability and clarity for the reader. As a result, in revised Figure 1 we do not longer depict LAMP1 (red channel 594 nm), We depict nuclei stained with DAPI, ACE2, PRCP, and PREP stained with IgG Alexa Fluor™ Plus 488 (green channel) and the merge of DAPI and 488 (see below).

We changed the Method section 3 accordingly, and it reads now on page 6, lines 140-152: “Cells were incubated with primary antibody polyclonal rabbit anti-ACE2 (AB_2792286, cat.no. PA5-85139, Invitrogen, Rockford, IL, US) diluted 1:100, polyclonal rabbit anti-PRCP (AB_1855676, cat. no. HPA 017065, BIOZOL, Eching, Germany) diluted 1:1000, or polyclonal rabbit anti-PREP (AB_2900433, PA5-115798, Invitrogen, Rockford, IL, US) diluted 1:200 for 1 h at room temperature. After three washing steps, secondary antibody polyclonal goat anti-rabbit IgG Alexa Fluor™ Plus 488 (AB_2633280, cat. no. A32731, Invitrogen, Rockford, IL, US) diluted 1:2000 was added and incubated for 1 h at room temperature in darkness.”

We also deleted the information concerning LAMP1 co-localization from the Results section and figure legend of revised Figure 1.

**Fig 1. Characterization of ACE2, PRCP, and PREP on protein level in hPC conducted by immunofluorescence.** Representative immunofluorescence images of (A) ACE2, (B) PRCP, and (C) PREP, demonstrating protein expression on hPC. (D) Control without primary antibody revealed no specific binding of secondary antibody goat anti-rabbit Alexa Fluor™ Plus 488. Nuclei were stained using DAPI (blue). Scale bar 50 µm.

As Reviewer 1 raised concerns about the expression and presence of the enzymes in hPC, we additionally performed western blot analyses of ACE2 and PRCP to provide further evidence. The data are added to the revised Supplement as “Method S2 Western Blot” and Supplement Figure S4. For more detailed information please see Reviewer 1 - Comment 1.6.

Comment 6
6. The authors should make more efforts to elaborate a little on each of their main results and their implications. Otherwise, the reader has to wait until the Discussion section to gain insight into the current work. This will enhance the readability of the manuscript by making it more engaging.

Response: We thank the Reviewer for this valuable comment. As suggested, we added more detailed information on the results’ implications within the Results section for a better understanding and readability as follows:

1. On page 16 lines 364-366: “Inhibitor assays revealed that Ang-(1–7) formation from the substrate Ang II was mediated by ACE2 and PRCP, but not by PREP.”
2. On page 16 lines 381-382: “These results suggest a higher enzyme activity and Ang II to Ang-(1-7) conversion capacity upon FFSS in hPC.”
3. On page 17 lines 397-398: “Overall, the enzymes involved in Ang II to Ang-(1-7) conversion are only moderately regulated upon FFSS.”
4. On page 19 lines 448-451: “In summary, in cell lysates, Ang-(1-7) formation by ACE2 was low, and most of the conversion was mediated by PRCP. Increased Ang-(1-7) formation rate upon FFSS cannot be attributed to ACE2 and PRCP, but rather indicates that another enzyme is involved.”
5. On page 20 lines 478-480:” Taken together, Ang-(1-7) formation rate in supernatants is predominantly driven by ACE2 but contribution of PRCP increases upon FFSS suggesting increased enzyme transport to the cell surface through exocytosis.”

Comment 7
7. Consider incorporating Fig. S2(A-D) part of the main figures because of their importance to understanding the results of this study.

Response: We thank the Reviewer for the important comment regarding clarity and readability. As suggested, we added Fig. 2S (A-D) to the revised main text as revised Fig. 2. Figure S2E and F of the original manuscript were changed to Figure 2SA and B in the revised Supplementary.

References

1.	Skidgel RA, Erdos EG. Cellular carboxypeptidases. *Immunol Rev.* 1998;**161**:129–41.

2.	Friedrich C, Endlich N, Kriz W, Endlich K. Podocytes are sensitive to fluid shear stress in vitro. *Am J Physiol Renal Physiol.* 2006;**291**(4):F856–65.

3.	Srivastava T, McCarthy ET, Sharma R, Cudmore PA, Sharma M, Johnson ML, et al. Prostaglandin E(2) is crucial in the response of podocytes to fluid flow shear stress. *J Cell Commun Signal.* 2010;**4**(2):79–90.

4.	Yang SH, Choi JW, Huh D, Jo HA, Kim S, Lim CS, et al. Roles of fluid shear stress and retinoic acid in the differentiation of primary cultured human podocytes. *Exp Cell Res.* 2017;**354**(1):48–56.

5.	Blaine J, Dylewski J. Regulation of the Actin Cytoskeleton in Podocytes. *Cells.* 2020;**9**(7).

6.	Donoghue M, Hsieh F, Baronas E, Godbout K, Gosselin M, Stagliano N, et al. A novel angiotensin-converting enzyme-related carboxypeptidase (ACE2) converts angiotensin I to angiotensin 1-9. *Circ Res.* 2000;**87**(5):E1–9.

7.	Wysocki J, Batlle D. ACE2 in the Urine: Where Does It Come From? *Kidney360.* 2022;**3**(12):2001–4.

8.	Clarke NE, Turner AJ. Angiotensin-converting enzyme 2: the first decade. *Int J Hypertens.* 2012;**2012**:307315.

9.	Rouaud F, Mean I, Citi S. The ACE2 Receptor for Coronavirus Entry Is Localized at Apical Cell-Cell Junctions of Epithelial Cells. *Cells.* 2022;**11**(4).

10.	Marquez E, Riera M, Pascual J, Soler MJ. Albumin inhibits the insulin-mediated ACE2 increase in cultured podocytes. *Am J Physiol Renal Physiol.* 2014;**306**(11):F1327–34.

11.	Lin H, Geurts F, Hassler L, Batlle D, Mirabito Colafella KM, Denton KM, et al. Kidney Angiotensin in Cardiovascular Disease: Formation and Drug Targeting. *Pharmacol Rev.* 2022;**74**(3):462–505.

12.	Balcarek J, Seva Pessoa B, Bryson C, Azizi M, Menard J, Garrelds IM, et al. Multiple ascending dose study with the new renin inhibitor VTP-27999: nephrocentric consequences of too much renin inhibition. *Hypertension.* 2014;**63**(5):942–50.

13.	Fisher ND, Jan Danser AH, Nussberger J, Dole WP, Hollenberg NK. Renal and hormonal responses to direct renin inhibition with aliskiren in healthy humans. *Circulation.* 2008;**117**(25):3199–205.

14.	Lawrence AC, Evin G, Kladis A, Campbell DJ. An alternative strategy for the radioimmunoassay of angiotensin peptides using amino-terminal-directed antisera: measurement of eight angiotensin peptides in human plasma. *J Hypertens.* 1990;**8**(8):715–24.

15.	Campbell DJ, Lawrence AC, Towrie A, Kladis A, Valentijn AJ. Differential regulation of angiotensin peptide levels in plasma and kidney of the rat. *Hypertension.* 1991;**18**(6):763–73.

16.	Campbell DJ, Kladis A, Valentijn AJ. Effects of losartan on angiotensin and bradykinin peptides and angiotensin-converting enzyme. *J Cardiovasc Pharmacol.* 1995;**26**(2):233–40.

17.	Roksnoer LC, van Veghel R, de Vries R, Garrelds IM, Bhaggoe UM, Friesema EC, et al. Optimum AT1 receptor-neprilysin inhibition has superior cardioprotective effects compared with AT1 receptor blockade alone in hypertensive rats. *Kidney Int.* 2015;**88**(1):109–20.

18.	Ye M, Wysocki J, William J, Soler MJ, Cokic I, Batlle D. Glomerular localization and expression of Angiotensin-converting enzyme 2 and Angiotensin-converting enzyme: implications for albuminuria in diabetes. *J Am Soc Nephrol.* 2006;**17**(11):3067–75.

19.	Hamming I, Timens W, Bulthuis ML, Lely AT, Navis G, van Goor H. Tissue distribution of ACE2 protein, the functional receptor for SARS coronavirus. A first step in understanding SARS pathogenesis. *J Pathol.* 2004;**203**(2):631–7.

20.	Schindelin J, Arganda-Carreras I, Frise E, Kaynig V, Longair M, Pietzsch T, et al. Fiji: an open-source platform for biological-image analysis. *Nat Methods.* 2012;**9**(7):676–82.

21.	Shariat-Madar Z, Mahdi F, Schmaier AH. Recombinant prolylcarboxypeptidase activates plasma prekallikrein. *Blood.* 2004;**103**(12):4554–61.
