# Supplementary material for: Upregulation of angiotensin-(1–7) formation in human podocytes – enzyme activity assay upon fluid flow shear stress
Source: PLoS One. 2026 Jan 9;21(1):e0339874. doi: 10.1371/journal.pone.0339874 (PMC12788633; doi:10.1371/journal.pone.0339874)
Supplement: Attachment — Submitted filename: Response to Reviewers-2.docx [file pone.0339874.s014.docx]

**PONE-D-25-20067R1 – Revision**

**Response to Reviewers and Editors**

**Response to Editors**

We thank the Editor for the thorough evaluation of our manuscript. In the following, we address the general remarks provided:

Journal Requirements:

If the reviewer comments include a recommendation to cite specific previously published works, please review and evaluate these publications to determine whether they are relevant and should be cited. There is no requirement to cite these works unless the editor has indicated otherwise.

Response: Thank you for the advice. However, the Reviewers did not propose any specific references.

Please review your reference list to ensure that it is complete and correct. If you have cited papers that have been retracted, please include the rationale for doing so in the manuscript text, or remove these references and replace them with relevant current references. Any changes to the reference list should be mentioned in the rebuttal letter that accompanies your revised manuscript. If you need to cite a retracted article, indicate the article’s retracted status in the References list and also include a citation and full reference for the retraction notice.

Response: We carefully reviewed our reference list and checked for retracted articles. We did not find any retracted article in our list. However, reference number 65 *(Velez JC, Bland AM, Arthur JM, Raymond JR, Janech MG. Characterization of renin-angiotensin system enzyme activities in cultured mouse podocytes. Am J Physiol Renal Physiol. 2007;293(1): F398–407. Epub 20070411. doi: 10.1152/ajprenal.00050.2007. PubMed PMID: 17429035.)* was corrected in 2008, as the authors had mistakenly labeled a measured peptide as Ang-(1-9), which was later found to be Ang-(2-10) [1]. The correction does not affect our citation of the study. Therefore, we have retained this reference.

We also identified an updated PMID number for reference number 2 and updated it in our reference list *(Inagami T. The renin-angiotensin system. Essays Biochem. 1994; 28:147–64. PubMed PMID: 7925317)*.

**Response to Review Comments to the Author**

**Response to Reviewer #2: (No Response)**

Response: We are glad that we were able to successfully address all of Reviewer’s #2 questions and comments.

**Response to Reviewer #3:**

Due to limitations in study design some comments could not be responded successfully.

Response: We thank the Reviewer #3 for carefully reading our manuscript and evaluating our responses to the Reviewers #1 and #2. Your comments helped us further improve our manuscript.

As a general note, all page and line numbers in our responses refer to the latest revised and marked manuscript version titled *“Revised Article with Changes Highlighted”*, unless otherwise specified.

Comment 1.1
This comment has been partially addressed.

Response: We thank the author for this comment and noticed that we need to explain the methodology in more detail.

a. What was the composition of the buffer used for the preparation? did it have any detergents? ionic strength?

Response: We used PBS (pH 7.4) to assess Ang-(1-7) formation in hPC cell lysates. Cells were scraped from the slides and transferred to PBS prior to sonification as described in the original manuscript on page 9 lines 220-221:” Cells were scratched from slides and transferred to a vial containing PBS.” We added the product information when first mentioned in the manuscript Method section – immunofluorescence in lines 133-134, where it now reads: “Medium was discarded and cells washed twice with phosphate buffered saline (PBS, cat. no. 14190094, Thermo Fisher Scientific, Waltham, MA, US).”

No detergents were used in the sample preparation procedure of cell lysates. To calculate the ionic strength, we relied on the manufacturer's specifications for the salt composition of PBS, assumed an ideal diluted solution and that all phosphorous compounds are dissociated completely. The resulting ionic strength for PBS at pH 7.4 is 166 mM.

b. Cells suspensions were sonicated for complete dissolution... I think complete dissolution of membranes is not possible with this methodology.

Response: We thank the reviewer for this comment. Cells were lysed using Vibra-Cell™ and membranes were destroyed by sonication using this methodology. We agree with the Reviewer, that the word “complete” cannot be used with absolute certainty in this context and therefore, deleted it from the sentence. The revised manuscript now reads in lines 221-223: “Cell suspensions were sonicated for dissolution using Vibra-Cell™ (Sonics & Materials Inc., Newtown, CT; US) and centrifugated at 16260 rcf for 10 min at 4°C.”

c. Was there any centrifugation step involved?
What was the duration and conditions of centrifugation? This is important to know what type of membranes author worked with. If no centrifugation was used the protocol seems not appropriate.

Response: That is a valuable comment. Indeed, we performed a centrifugation step. We sincerely apologize for our inaccuracy in the description of the methodology. For the cell lysates sample preparation, samples were centrifuged at 4°C for 10 minutes and 16260 rcf after sonication. Supernatants were collected and protein concentration quantified. We have amended the methodology in the revised manuscript. It now reads in lines 221-226: “Cell suspensions were sonicated for dissolution using Vibra-Cell™ (Sonics & Materials Inc., Newtown, CT; US) and centrifugated at 16260 rcf for 10 min at 4°C. Supernatants were collected and normalized to total protein concentration determined using BIO-RAD Protein Assay Dye with bovine serum albumin as protein standard (cat. no. 5000006, Hercules, CA, US).”

Comment 1.3
This comment has not been addressed. Authors postulate now the participation of another enzyme that may contribute to the increased Ang-(1-7) formation observed under FFSS in cell lysates. Which enzyme do authors considered?

Response: We recognize that further consideration and clarification are needed regarding additional potential enzymes involved in Ang-(1-7) formation, particularly as Reviewer #4 has raised a similar concern. We agree that at least a literature-based hypothesis, as suggested by Reviewer #4, is necessary to address this mechanistic gap. Thus, we conducted an extensive literature search to identify enzymes that could potentially account for the observed conversion of Ang II to Ang-(1-7) besides ACE2, PRCP and PREP:

Cathepsin A (CTSA), a lysosomal enzyme widely expressed in human tissues including the kidney [2], was initially proposed as a candidate for Ang-(1-7) formation. Early studies by Grynbaum and Marks reported that Cathepsin A from rat brain could release the C-terminal Phe from Ile^5-Ang II, producing Ang-(1-7) [3]. In contrast, Miller et al. found that Cathepsin A from porcine kidney cleaved Ang I to Ang-(1-9) and to Ang II, but did not hydrolyze Ang II [4]. Subsequent studies similarly highlighted Ang I as a primary substrate of Cathepsin A [5].

Grobe et al. reported that nephrolysin (NEP), thimet oligopeptidase (THOP1), and neurolysin (NLN) can generate Ang-(1-7), though they cleave Ang I and not Ang II [6]. Both NLN and THOP1 cleave Ang I at the Pro-Phe bond to form Ang-(1-7) [7, 8]. NLN is ubiquitously expressed, including in the kidney [9], but is primarily studied in the brain, where it is associated with stroke [10, 11]. While NLN can inactivate Ang II [8, 11, 12], this produces Ang-(1–4), not Ang-(1–7) [13]. Consequently, it is not a potential enzyme for our investigated conversion of Ang II to Ang-(1-7).

Velez et al. suggested that THOP1 may also convert Ang II to Ang-(1-7), alongside ACE2, RPCP, and PREP [14]. Early assays found no Ang II cleavage by recombinant rat THOP1 [15], while later studies suggested inefficient cleavage due to low catalytic efficiency [16]. Since existing statements are contradictory, we conclude that it remains unclear whether THOP1 actually hydrolyzes Ang II besides Ang I. Furthermore, no studies have identified specific Ang II–derived products of THOP1. Thus, THOP1 might inactivate Ang II by generating Ang-(1–4) or it might produce Ang-(1–7).

Overall, current evidence does not conclusively show that any of these enzymes convert Ang II to Ang-(1–7). Further studies are needed to define the enzymes regulating the RAS in podocytes, particularly experimentally assessing THOP1 and Cathepsin A under our conditions in hPC. We addressed these considerations in the revised Discussion section, which now reads in lines 522-533: “Additional enzymes involved in the degradation of angiotensin peptides that should be further investigated include Cathepsin A (CTSA; EC 3.4.16.5) and thimet oligopeptidase (THOP1; EC 3.4.24.15). Although CTSA is mainly known to hydrolyze Ang I [79], early studies suggest it may also cleave Ang II to Ang-(1–7) [80]. THOP1 is well established in cleaving Ang I to Ang-(1–7) [81,82], and further studies have indicated that it can also cleave Ang II, albeit inefficiently due to its low catalytic efficiency [83]. However, potential Ang II–derived cleavage products of THOP1 have not yet been identified. Future research might therefore specifically examine THOP1 and Cathepsin A under the experimental conditions in hPC to clarify their potential roles in local RAS regulation and defining more precisely the enzymatic mechanisms underlying Ang II processing in hPC.”

Comment 1.4
I concur with Reviewer 1 int that Ang II levels used were too high to make an extrapolation to in vivo settings

Response: We thank the Reviewer for raising this concern once again. As noted in our response to Comment 1.4 in our first revision, we agreed and acknowledged that the concentration of Ang II used in our experiments does not reflect in vivo peptide levels and represents a limitation of the study. To more clearly emphasize the importance of this comment, we have highlighted this limitation explicitly in the discussion section of the newly revised manuscript. It now reads in lines 604-610: “We deliberately chose a high Ang II concentration of 1µg/mL to evaluate catalytic capacities under standardized conditions avoiding artificial limitations on enzyme activity. Although the exogenously applied Ang II concentration does not reflect physiological in vivo peptide levels, this approach enabled us to use spiked Ang II as a substrate, allowing clear identification of only the spiked product via mass spectrometry and the precise calculation of enzyme activity.”

Comment 1.15
ACE2 activity coming from the cell surface cannot be evaluated with this protocol since all membranes were mix together after sonication and centrifugation of lysates

Response: Two different experimental setups were used to assess enzyme activity in cell lysates and on the cell surface. For cell lysate measurements, cells were sonicated as described in the Method section “Cell lysates sample preparation”. Enzyme activities at the cell surface were determined using intact cells as described in the Method section “Cell supernatants sample preparation”, where Ang-(1-7) formation was measured in supernatants of intact cells. To highlight the use of cell supernatants we wrote in the revised manuscript in lines 238-241: “100µL inhibitor solutions were applied each into one chamber of the cassette onto hPC and incubated for 1 h at 37 °C. Supernatants were collected and, following stabilization by acidification, Ang-(1-7) formation was quantified by LC-MS/MS (Methods 7.3).”

**Response to Reviewer #4:**

This study clarifies how fluid shear stress (FFSS) regulates Angiotensin-(1-7) formation in human podocytes. The authors show that the intracellular enzyme PRCP, not ACE2, is the dominant source of Ang-(1-7) production, a potentially protective pathway enhanced by FFSS. Here, a few concerns regarding data interpretation and discussion need to be addressed.

Response: We appreciate the Reviewer’s overall feedback, as well as his thorough and thoughtful comments provided below. They have been extremely valuable and we hope that we have addressed them sufficiently.

As a general note, all page and line numbers in our responses refer to the latest revised and marked manuscript version titled *“Revised Article with Changes Highlighted”*, unless otherwise specified.


1. The residual Ang-(1-7) formation in hPC lysates after dual ACE2 and PRCP inhibition (Fig 5) is noted to be upregulated by FFSS. This finding is a key mechanistic gap, the authors hypothesize another enzyme is involved but offer no data. Experimental or literature-based discussion is essential to hypothesize which other peptidases (e.g., Cathepsin A) could account for this activity, thereby providing a complete map of the RAS balance in the podocyte and guiding future research.

Response: We thank the Reviewer for this valuable comment and acknowledge the importance of further elaborating on additional peptidases involved in Ang-(1-7) formation. We conducted an extensive literature search to identify enzymes that might account for the observed conversion of Ang II to Ang-(1-7).

Cathepsin A (CTSA), a lysosomal enzyme widely expressed in human tissues including the kidney [2], was initially proposed as a candidate for Ang-(1-7) formation. Early studies by Grynbaum and Marks reported that Cathepsin A from rat brain could release the C-terminal Phe from Ile^5-Ang II, producing Ang-(1-7) [3]. In contrast, Miller et al. found that Cathepsin A from porcine kidney cleaved Ang I to Ang-(1-9) and to Ang II, but did not hydrolyze Ang II [4]. Subsequent studies similarly highlighted Ang I as a primary substrate of Cathepsin A [5].

Grobe et al. reported that nephrolysin (NEP), thimet oligopeptidase (THOP1), and neurolysin (NLN) can generate Ang-(1-7), though they cleave Ang I and not Ang II [6]. Both NLN and THOP1 cleave Ang I at the Pro-Phe bond to form Ang-(1-7) [7, 8]. NLN is ubiquitously expressed, including in the kidney [9], but is primarily studied in the brain, where it is associated with stroke [10, 11]. While NLN can inactivate Ang II [8, 11, 12], this produces Ang-(1–4), not Ang-(1–7) [13]. Consequently, it is not a potential enzyme for our investigated conversion of Ang II to Ang-(1-7).

Velez et al. suggested that THOP1 may also convert Ang II to Ang-(1-7), alongside ACE2, RPCP, and PREP [14]. Early assays found no Ang II cleavage by recombinant rat THOP1 [15], while later studies suggested inefficient cleavage due to low catalytic efficiency [16]. Since existing statements are contradictory, we conclude that it remains unclear whether THOP1 actually hydrolyzes Ang II besides Ang I. Furthermore, no studies have identified specific Ang II–derived products of THOP1. Thus, THOP1 might inactivate Ang II by generating Ang-(1–4) or it might produce Ang-(1–7).

Overall, current evidence does not conclusively show that any of these enzymes convert Ang II to Ang-(1–7). Further studies are needed to define the enzymes regulating the RAS in podocytes, particularly experimentally assessing THOP1 and Cathepsin A under our conditions in hPC. We addressed these considerations in the revised Discussion section, which now in lines 522-533: “Additional enzymes involved in the degradation of angiotensin peptides that should be further investigated include Cathepsin A (CTSA; EC 3.4.16.5) and thimet oligopeptidase (THOP1; EC 3.4.24.15). Although CTSA is mainly known to hydrolyze Ang I [79], early studies suggest it may also cleave Ang II to Ang-(1–7) [80]. THOP1 is well established in cleaving Ang I to Ang-(1–7) [81,82], and further studies have indicated that it can also cleave Ang II, albeit inefficiently due to its low catalytic efficiency [83]. However, potential Ang II–derived cleavage products of THOP1 have not yet been identified. Future research might therefore specifically examine THOP1 and Cathepsin A under the experimental conditions in hPC to clarify their potential roles in local RAS regulation and defining more precisely the enzymatic mechanisms underlying Ang II processing in hPC.”

2. This study's conclusion rests on the idea that the PRCP-driven Ang-(1-7) increase is a "renoprotective" shift in the RAS balance, but this is never demonstrated functionally in the model. Determine if PRCP inhibition significantly exacerbates this FFSS-induced injury, thereby validating its protective role.

Response: We thank the Reviewer for this important comment. It is correct that our conclusion that increased Ang-(1-7) formation may exert renoprotective effects has not been functionally demonstrated. Rather, our hypothesis is based on the extensive literature describing the signaling pathways and biological effects of Ang-(1-7). Previously, renal PRCP activity has been functionally linked to the pathogenesis of renovascular hypertension in two-kidney, one-clip Goldblatt hypertensive mice [17].

We agree with the Reviewer that the impact of PRCP inhibition on FFSS-induced damage would be an important step to further validate the potential protective properties of PRCP in our experimental setting. However, this would require a whole set of additional studies aiming to validate PRCP inhibition in our experimental system beyond the scope of this initial hypothesis generating study. Nevertheless, we added this important aspect in the revised Discussion section, where it now reads in lines 598-601: “Further research is needed to determine if the proposed renoprotective role of PRCP can be functionally demonstrated, for instance by investigating whether PRCP inhibition exacerbates FFSS-induced damage, or whether PRCP overexpression or enhanced activity mitigates FFSS-induced injury.”

3. The authors noted the discrepancy: PRCP mRNA was slightly reduced while total Ang-(1-7) formation (driven by PRCP in lysate) was increased and hypothesized this was due to post-translational modifications. Perform quantitative densitometry on the existing Western Blot data (S4 Fig) for ACE2 and PRCP to confirm that the observed changes in enzyme activity are independent of changes in total protein mass.

Response: We agree with the Reviewer that, in order to make a well-supported statement, densitometric analysis of western blot data from both control and FFSS samples would be required. However, the western blots shown in S4 Fig were performed specifically in response to Reviewer #1 comment 1.6, which questioned the presence of PRCP and ACE2 at the protein level. Consequently, these blots were generated using only untreated hPC. Densitometric analysis of the data presented in S4 Fig would therefore not address the Reviewer’s current question.

To properly answer this comment, a completely new panel of FFSS experiments would need to be conducted, followed by protein isolation from FFSS-treated and control samples and subsequent western blot analysis beyond the scope of this hypothesis generating initial study.

4. They established the significance of Ang-(1-7) from the literature but did not functionally validate that the PRCP-driven increase specifically confers protection in their FFSS model. Adapt the Discussion to explicitly link the observed PRCP activation under FFSS with the known molecular protective signaling of Ang-(1-7), framing this as the key translational hypothesis.

Response: We appreciate the Reviewer’s comment and agree that the functional link between PRCP-driven increase in Ang-(1-7) formation and protective signaling is a central translational question. While direct functional validation of Ang-(1-7) signaling was beyond the scope of the present study, our data provide a mechanistically coherent rationale that aligns with well-established literature on protective Ang-(1-7) signaling in podocytes. In response to the Reviewer’s suggestion, we have revised the Discussion to more explicitly articulate this connection.

In the revised text, we highlight that PRCP represents the major enzymatic contributor to Ang II–to–Ang-(1–7) conversion in our experiments. We now integrate this finding with the molecular mechanisms of Ang-(1–7), emphasizing that the predominance of PRCP in this processing pathway positions the enzyme as a relevant determinant of Ang-(1–7) availability in the context of FFSS. We adapted our Discussion section accordingly. It now reads in lines 556-590:” In our study, PRCP is the predominant enzymatic contributor to Ang-(1-7) generation in podocyte lysates. PRCP cleaves carboxy-terminal residues from peptides with a penultimate proline, including Ang II and Ang III [78, 84-86]. Its relevance for renal homeostasis is supported by findings that renal PRCP expression is reduced in hypertensive rats and restored by losartan [87], and that PRCP depletion in mice induced endothelial dysfunction, hypertension, alterations in renal and vascular eNOS, and increased ROS formation [88]. While FFSS did not directly activate PRCP, this enzymatic capacity provides a plausible mechanism to shift the local balance from Ang II toward Ang-(1‑7). This is particularly relevant because Ang-(1-7) is well known to oppose Ang II–mediated injury by exerting vasodilatory, antioxidative, antiproliferative, antifibrotic, and anti-inflammatory actions [17].

Mechanical stress, including FFSS, is known to disrupt podocyte structure and survival by inducing cytoskeletal remodeling, detachment, and apoptosis, partly through mechanotransduction pathways involving Akt–GSK3β–β-catenin and MAPK/ERK signaling [24, 93, 94]. It has also been reported, that mechanical stress activates the local RAS, enhancing Ang II production and its downstream MAPK, TGF-β, and PI3K cascades, thereby reducing nephrin expression and promoting apoptosis and cytoskeletal destabilization [31, 43, 95, 96]. Given these well-established Ang II-mediated injury mechanisms, a FFSS-associated shift toward Ang-(1-7) could be renoprotective and counterbalance FFSS-induced damage. Moreover, Ang-(1-7) has been demonstrated to directly signal protective in podocytes. It restored nephrin, podocin, and WT1 levels and reduced apoptosis under high-glucose conditions in mouse podocytes. These effects were blocked by the MAS1 receptor antagonist A-779 [92D. In human podocytes exposed to preeclampsia serum, Ang-(1-7) suppressed phosphorylation of p38, ERK1/2, and JNK in a MAS1 receptor-dependent manner, indicating inhibition of stress-activated MAPK pathways [91]. Ang-(1-7) also stimulated modest AT₂R-dependent nitric oxide production [90], a mediator of podocyte redox balance and cytoskeletal regulation [97]. The renoprotective effects of Ang-(1-7) extend to an in vivo mouse model, where cyclic Ang-(1-7) limited albuminuria progression, podocyte dysfunction, and glomerular fibrosis similarly to ACE inhibition [89].

The predominant contribution of PRCP to Ang-(1–7) formation supports the translational hypothesis that PRCP may shape how hPC invoke Ang-(1–7)–dependent protective signaling under FFSS, thereby counteracting Ang II– and shear stress–induced injury.”

In addition, we restructured the Discussion section to improve readability while incorporating our responses to your valuable requests:

- Discussion of inhibitory test were deleted in lines 485-494 and moved upward in lines 464-473.
- The sentence “In this study, we characterized the enzymes involved in conversion of Ang II to Ang-(1-7) in hPC upon FFSS, using pharmacological inhibition of the potentially involved converting enzymes and LC/MS-MS analysis.” was moved from lines 414-416 to 450-452.
- The sentence “Our results revealed an increased conversion of Ang II to Ang-(1-7) in hPC lysates exposed to FFSS. This was primarily driven by PRCP.” was moved from lines 416-418 to lines 517-518 with slight modification. It now reads: “In contrast to supernatants, our results reveal an increased conversion of Ang II to Ang-(1-7) in hPC lysates exposed to FFSS which was primarily driven by PRCP.”

5. Briefly justify the physiological relevance of the 1 dyne/cm2 for 2 h FFSS parameters in the Discussion to confirm they model pathological hyperfiltration stress.

Response: We included a brief explanation in the Discussion section justifying the FFSS parameters, as requested by the Reviewer. It now reads in lines 452-463: “FFSS parameters to model pathological hyperfiltration stress were used based on previous research. FFSS at the podocyte surface in healthy adult mice has been estimated to be approximately 0.3 dyne/cm² [24]. In models of unilateral nephrectomy reflecting a hyperfiltrating state, FFSS on mouse podocytes increases by 1.5–2.5-fold, reaching ~0.5–0.75 dyne/cm² [21]. As reported by others, applying 0.5 dyne/cm² on human primary podocytes promoted expression of podocyte-specific markers, supporting a physiological relevance of this stress level in hPC [27]. Based on these data, we selected 1 dyne/cm² for 2 hours to model pathological hyperfiltration, representing a mild but sustained increase over normal shear stress, sufficient to induce mechanosensitive responses in glomerular cells without causing acute cell detachment and loss.”

6. The authors observed low ACE2 contribution in the cell lysates but high ACE2 contribution in the supernatant. Revise the Discussion and explicitly clarify how the differential compartmentalization (membrane vs. intracellular) accounts for the highly distinct enzymatic roles of ACE2 and PRCP across the two assay types.

Response: This is a valuable comment, which helped us to clarify our Discussion. As suggested by the Reviewer, we have elaborated on the cellular compartmentalization and distribution of PRCP and ACE2 as well as their enzymatic role. It now reads in the Discussion section lines 501-509: “In the present study, we assessed the metabolism of Ang II to Ang-(1-7) in hPC cell lysates and on the cell surface, and evaluated the respective contributions of ACE2 and PRCP. On the cell surface, ACE2 activity was found to play a more prominent role in Ang-(1-7) formation than PRCP, especially under control conditions. This is consistent with ACE2 being predominantly localized at the plasma membrane, where it cleaves Ang II in the ultrafiltrate within Bowman’s space [64, 70-73]. In hPC lysates, Ang-(1–7) formation was predominantly driven by PRCP, reflecting both its higher expression levels compared to ACE2 and its mainly lysosomal localization, which enables it to cleave and degrade peptides intracellularly [74-76].”

References

1.	Corrigendum. American Journal of Physiology-Renal Physiology. 2008;295(3):F857–F. doi: 10.1152/ajprenal.zh2-5280-corr.2008.

2.	Timur ZK, Akyildiz Demir S, Seyrantepe V. Lysosomal Cathepsin A Plays a Significant Role in the Processing of Endogenous Bioactive Peptides. Front Mol Biosci. 2016;3:68. Epub 20161025. doi: 10.3389/fmolb.2016.00068. PubMed PMID: 27826550; PubMed Central PMCID: PMCPMC5078471.

3.	Grynbaum A, Marks N. Characterization of a rat brain catheptic carboxypeptidase (cathepsin A) inactivating angiotensin-II. J Neurochem. 1976;26(2):313–8. doi: 10.1111/j.1471-4159.1976.tb04482.x. PubMed PMID: 1255194.

4.	Miller JJ, Changaris DG, Levy RS. Conversion of angiotensin I to angiotensin II by cathepsin A isoenzymes of porcine kidney. Biochem Biophys Res Commun. 1988;154(3):1122–9. doi: 10.1016/0006-291x(88)90257-4. PubMed PMID: 3408489.

5.	Jackman HL, Massad MG, Sekosan M, Tan F, Brovkovych V, Marcic BM, et al. Angiotensin 1-9 and 1-7 release in human heart: role of cathepsin A. Hypertension. 2002;39(5):976–81. doi: 10.1161/01.hyp.0000017283.67962.02. PubMed PMID: 12019279.

6.	Grobe N, Weir NM, Leiva O, Ong FS, Bernstein KE, Schmaier AH, et al. Identification of prolyl carboxypeptidase as an alternative enzyme for processing of renal angiotensin II using mass spectrometry. Am J Physiol Cell Physiol. 2013;304(10):C945–53. Epub 20130207. doi: 10.1152/ajpcell.00346.2012. PubMed PMID: 23392115; PubMed Central PMCID: PMCPMC3651643.

7.	Rioli V, Kato A, Portaro FC, Cury GK, te Kaat K, Vincent B, et al. Neuropeptide specificity and inhibition of recombinant isoforms of the endopeptidase 3.4.24.16 family: comparison with the related recombinant endopeptidase 3.4.24.15. Biochem Biophys Res Commun. 1998;250(1):5–11. doi: 10.1006/bbrc.1998.8941. PubMed PMID: 9735321.

8.	Shi K, Bagchi S, Bickel J, Esfahani SH, Yin L, Cheng T, et al. Structural basis of divergent substrate recognition and inhibition of human neurolysin. Sci Rep. 2024;14(1):18420. Epub 20240808. doi: 10.1038/s41598-024-67639-w. PubMed PMID: 39117724; PubMed Central PMCID: PMCPMC11310207.

9.	Cavalcanti DM, Castro LM, Rosa Neto JC, Seelaender M, Neves RX, Oliveira V, et al. Neurolysin knockout mice generation and initial phenotype characterization. J Biol Chem. 2014;289(22):15426–40. Epub 20140409. doi: 10.1074/jbc.M113.539148. PubMed PMID: 24719317; PubMed Central PMCID: PMCPMC4140899.

10.	Checler F, Ferro ES. Neurolysin: From Initial Detection to Latest Advances. Neurochem Res. 2018;43(11):2017–24. Epub 20180829. doi: 10.1007/s11064-018-2624-6. PubMed PMID: 30159819.

11.	Rashid M, Wangler NJ, Yang L, Shah K, Arumugam TV, Abbruscato TJ, et al. Functional up-regulation of endopeptidase neurolysin during post-acute and early recovery phases of experimental stroke in mouse brain. J Neurochem. 2014;129(1):179–89. Epub 20131119. doi: 10.1111/jnc.12513. PubMed PMID: 24164478.

12.	Karamyan VT. The role of peptidase neurolysin in neuroprotection and neural repair after stroke. Neural Regen Res. 2021;16(1):21–5. doi: 10.4103/1673-5374.284904. PubMed PMID: 32788443; PubMed Central PMCID: PMCPMC7818881.

13.	Brown CK, Madauss K, Lian W, Beck MR, Tolbert WD, Rodgers DW. Structure of neurolysin reveals a deep channel that limits substrate access. Proc Natl Acad Sci U S A. 2001;98(6):3127–32. Epub 20010306. doi: 10.1073/pnas.051633198. PubMed PMID: 11248043; PubMed Central PMCID: PMCPMC30618.

14.	Velez JC. Prolyl carboxypeptidase: a forgotten kidney angiotensinase. Focus on "Identification of prolyl carboxypeptidase as an alternative enzyme for processing of renal angiotensin II using mass spectrometry". Am J Physiol Cell Physiol. 2013;304(10):C939–40. Epub 20130403. doi: 10.1152/ajpcell.00081.2013. PubMed PMID: 23552286; PubMed Central PMCID: PMCPMC3651640.

15.	Camargo AC, Gomes MD, Reichl AP, Ferro ES, Jacchieri S, Hirata IY, et al. Structural features that make oligopeptides susceptible substrates for hydrolysis by recombinant thimet oligopeptidase. Biochem J. 1997;324 ( Pt 2)(Pt 2):517–22. doi: 10.1042/bj3240517. PubMed PMID: 9182712; PubMed Central PMCID: PMCPMC1218460.

16.	Rioli V, Gozzo FC, Heimann AS, Linardi A, Krieger JE, Shida CS, et al. Novel natural peptide substrates for endopeptidase 24.15, neurolysin, and angiotensin-converting enzyme. J Biol Chem. 2003;278(10):8547–55. Epub 20021224. doi: 10.1074/jbc.M212030200. PubMed PMID: 12500972.

17.	Grobe N, Leiva O, Morris M, Elased KM. Loss of prolyl carboxypeptidase in two-kidney, one-clip goldblatt hypertensive mice. PLoS One. 2015;10(2):e0117899. Epub 20150223. doi: 10.1371/journal.pone.0117899. PubMed PMID: 25706121; PubMed Central PMCID: PMCPMC4338234.
